# Synthetic study of *vic*-bromination of diarylacetylenes, easy purification and separation

**DOI:** 10.3762/bjoc.22.61

**Published:** 2026-05-22

**Authors:** Akane Togo, Hiyono Suzuki, Yuto Akai, Makoto Matsumoto, Yoshinori Suzuma, Hidehiko Kodama, Kouichi Matsumoto

**Affiliations:** 1 Department of Chemistry, School of Science and Engineering, Kindai University, 3-4-1 Kowakae, Higashi-osaka, Osaka 577-8502, Japan; 2 Nippon Pharmaceutical Chemicals Co., Ltd., 2-8-18 Chodo, Higashi-osaka, Osaka 577-0056, Japan

**Keywords:** bromination, diarylacetylenes, green chemistry, purification and separation, stereoselective reaction

## Abstract

*E*-Selective bromination for diphenylacetylene was established by using the combination of NBS and FeBr_3_ in CH_2_Cl_2_. In addition, easy purification and separation from the crude product were found. When the crude product was treated with heptane, the *E* isomer could be recovered as a solid material by utilizing the difference in solubility. On the other hand, the *Z* isomer could be removed by filtration while remaining in solution.

## Introduction

**Scheme 1:**
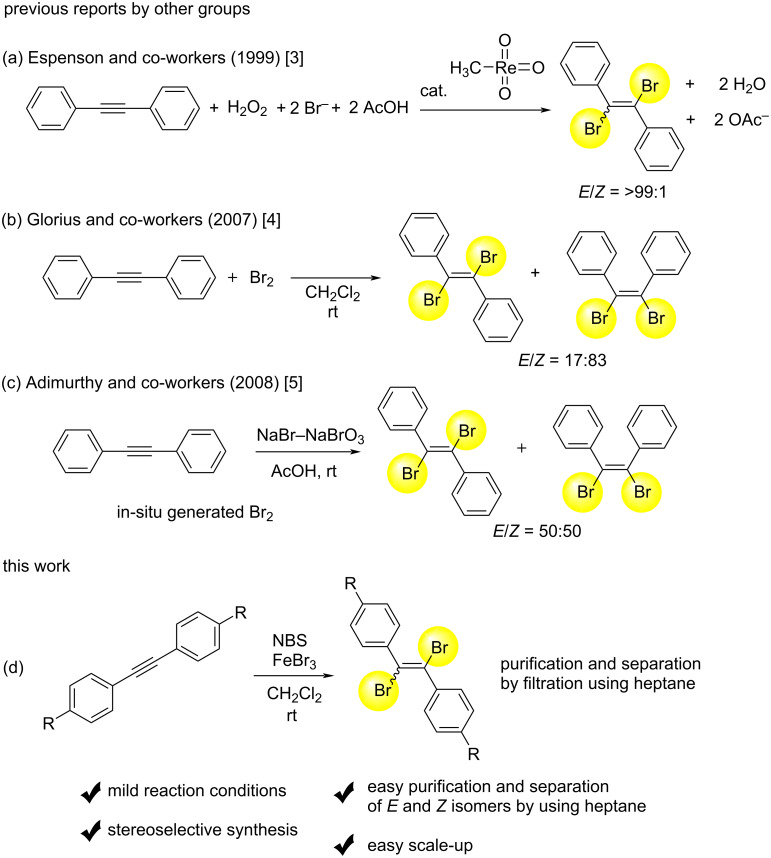
Selected and previous reports for bromination of diphenylacetylenes (a–c), and this work (d).

The addition reaction of bromine to diphenylacetylene is one of basic reactions and is recognized as important, because resulting 1,2-dibromo-1,2-diphenylethylene can serve as a precursor for various molecular transformations in organic synthesis [1–2]. This reaction seems to be simple, but some reported studies suggest that it is surprisingly difficult to obtain the *E* isomer selectively (Scheme 1). For selected examples, Espenson and co-workers reported in 1999 that *E*-selective bromination of diphenylacetylene took place by using H_2_O_2_, NaBr, and AcOH in the presence of MTO (methyltrioxorhenium(VII)) to give (*E*)-1,2-dibromo-1,2-diphenylethylene, selectively (Scheme 1a) [3]. However, MTO is an expensive reagent and a more versatile synthetic method is highly needed. As a simple method, the reaction of diphenylacetylene and Br_2_ in CH_2_Cl_2_ was reported by Glorius in 2007 [4], but the selectivity was reported for the *Z* isomer (Scheme 1b). As another example in 2008, Adimurthy and co-workers proposed the combination of NaBr-NaBrO_3_ in AcOH in order to prepare in-situ generated Br_2_, which was reacted with diphenylacetylene to give no selectivity between *E* and *Z* isomers of 1,2-dibromo-1,2-diphenylethylenes (Scheme 1c) [5]. Previous studies reported the selective synthesis of (*E*)-1,2-dibromo-1,2-diphenylethylene and related compounds, but Br_2_ or expensive reagent, which was difficult to handle, was often used [6–15].

Based on these research backgrounds, we have also been interested in the selective synthesis of the *E* isomer of 1,2-dibromo-1,2-diphenylethylene, together with simple purification and separation toward process chemistry, because the compound is an attractive synthetic intermediate for further transformations. During the course of our studies, we have found that the combination of NBS (*N*-bromosuccinimide) and FeBr_3_ in CH_2_Cl_2_ was good for the reaction with diphenylacetylene [16–19] to give 1,2-dibromo-1,2-diphenylethylene with high *E*-selectivity. The present method has some advantages. First, is mild reaction conditions, and second is stereoselective synthesis. In addition, we have found an easy purification and separation method of *E* and *Z* isomers of the crude product by using heptane. The method utilizes the difference in solubility of *E* and *Z* isomers. Because of this technique, the purification, separation, and scale-up were easy to perform. In this paper, we wish to report the details of the results.

## Results and Discussion

In order to obtain the *E* isomer of 1,2-dibromo-1,2-diphenylethylene, we have examined the reaction regarding optimized conditions (Table 1). The typical procedure is as follows: the reaction of diphenylacetylene (**1a**, 0.5 mmol) with NBS (*N*-bromosuccinimide, X equiv), FeBr_3_ (1.0 equiv), *n*-Bu_4_NBr (Y equiv), and TEMPO (2,2,6,6-tetramethylpiperidine 1-oxyl free radical, Z equiv) in solvent (2 mL) at the desired temperature was conducted for 23 h or 2 h. After the reaction, the typical work-up procedure was carried out and the crude product was analyzed for the ratio of *E* and *Z* isomers and starting material **1a**, by using HPLC. Purification and separation from the crude product was conducted by the filtration using heptane (Table 1). At first, we have recognized that ***E*-2a** is solid and ***Z*-2a** is liquid in heptane. After various considerations, we found that ***E*-2a** could be isolated by only filtration, utilizing the difference in solubility of ***E*-2a** and ***Z*-2a** in heptane from the crude product.

**Table 1:** Reaction optimization of bromination reaction of diphenylacetylene (**1a**).

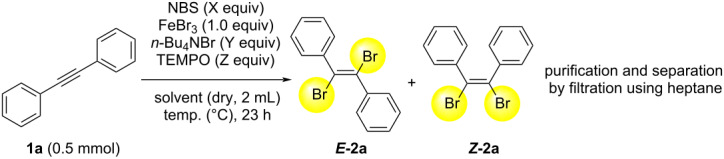							

Entry	NBS (X equiv)	*n*-Bu_4_NBr (Y equiv)	TEMPO (Z equiv)	Temp. (^o^C)	Solvent	Ratio of ***E*-2a**/***Z*-2a**/**1a**^a^	Yield (%) of ***E*-2a**^b^

1^c^	2.2	0	0	rt	CH_2_Cl_2_	86:14:0	78
**2** **^c,d^**	**2.2**	**0**	**0**	**rt**	**CH** **_2_** **Cl** **_2_**	**87:13:0**	**78**
3^d^	1.2	0	0	rt	CH_2_Cl_2_	88:12:0	79
4	2.2	0	1.0	rt	CH_2_Cl_2_	91:8:1	77
5^d^	2.2	0.5	1.0	rt	CH_2_Cl_2_	80:2:18	73
6^d^	2.2	1.0	1.0	rt	CH_2_Cl_2_	55:0:45	47
7	2.2	1.0	0.2	rt	CH_2_Cl_2_	88:12:0	79
8	2.2	1.0	0.5	rt	CH_2_Cl_2_	78:2:20	68
9^e^	2.2	1.0	0	rt	CH_2_Cl_2_	82:18:0	72
10	2.2	1.0	0.5	40	CH_2_Cl_2_	85:3:12	70
11	2.2	1.0	0.5	60	C_2_H_8_Cl_2_	72:3:25	63
12	2.2	0	0	rt	C_2_H_8_Cl_2_	58:42:0	54

^a^Calculated from the ratio of HPLC area for the crude product. ^b^Isolated yield of ***E*-2a** by filtration with heptane from the crude product. The yield of ***E-*2a** was obtained from the solids recovered after the filtration. ^c^Reaction time was 2 hours. ^d^Calculated from the ratio of GC area for the crude product. ^e^Reaction time was 5 hours. C_2_H_8_Cl_2_ = 1,2-dichloroethane.

In Table 1, entry 1, the combination of NBS (2.2 equiv) and FeBr_3_ (1.0 equiv) for 2 h gave the ratio of ***E*-2a**/***Z*-2a**/**1a** such as 86:14:0 by HPLC, in which ***E*-2a** was obtained in 78% yield. Similarly, to examine the reproducibility of the chemical yield and the ratio of ***E*-2a**/***Z*-2a**/**1a** by GC analysis, Table 1, entry 2 was shown. The reaction time of 2 h gave the same ratio of ***E*-2a**/***Z*-2a**/**1a** by GC analysis, and ***E-2a*** was obtained in 78% yield (Table 1, entry 2). The use of a smaller amount of NBS (1.2 equiv) did not result in a decrease of yield, indicating that FeBr_3_ is thought to be one of the sources of bromine (Table 1, entry 3). To increase the chemical yield and ratio of ***E*-2a**/***Z*-2a**/**1a**, various parameters such as NBS, *n*-Bu_4_NBr, TEMPO, temperature, and solvent were examined (Table 1, entries 3–12). Because there was a possibility that a bromine radical was involved, TEMPO was used for the purpose of the ratio-control of *E* and *Z* isomers. TEMPO was found to have a tendency to suppress the generation of ***Z*-2a** in CH_2_Cl_2_ (Table 1, entries 6 and 8) [20]. Among Table 1, entries 5–11, the addition of *n*-Bu_4_NBr did not seem to have any effect. Instead of CH_2_Cl_2_, C_2_H_8_Cl_2_ (1,2-dichloroethane) was used as the solvent, but the yield of ***E*-2a** was decreased (Table 1, entries 11 and 12). Generally, decomposition of the product or over reaction was not observed by GC and/or GC–MS analysis, in the case of the longer reaction time of 23 h. We have examined other Lewis acids such as ZnBr_2_, LiBr, and FeBr_2_ instead of the use of FeBr_3_ under similar reaction conditions. The use of ZnBr_2_ for reaction time of 4 h showed a ratio of ***E*-2a**/***Z*-2a**/**1a** = 86:14:0 by HPLC analysis and 68% yield of ***E*-2a**. In the case of LiBr, the desired reaction progressed only slightly, even after two days, and the ratio of ***E*-2a**/***Z*-2a**/**1a** = 17:3:80 was indicated by GC analysis. In addition, the use of FeBr_2_ for a reaction time of 3 h gave an ***E*-2a**/***Z*-2a**/**1a** ratio of 67:13:20 by GC analysis and 57% yield of ***E*-2a**. Based on these investigations, we have adopted the conditions of Table 1, entry 2, because better chemical yield of ***E*-2a** as well as a higher ratio of ***E*-2a**/***Z*-2a**/**1a** with short reaction time were attractive for chemical synthesis in the laboratory [21].

Next, we have investigated the scope and limitations by using various diarylacetylenes bearing the substituents on the benzene ring at *para*-position. The results were summarized in Table 2. Depending on the product's characteristics, either heptane or methanol was used for purification and separation. The reaction of 1,2-di-*p*-tolylethyne (**1b**) under the optimized conditions gave the corresponding product with a ratio of 68:32 of *E* and *Z* isomers. The isolated yield of ***E*-2b** was <54% yield (Table 2, entry 1). Likewise, 1,2-bis(4-(*tert*-butyl)phenyl)ethyne (**1c**) showed the similar tendency to give ***E*-2c** in 45% yield (Table 2, entry 2). Poor selectivity as shown in entries 1 and 2 might be due to the stability of the intermediate. In the cases of Table 2, entries 3–6 residues such as MeO, F, Br and CN as the substituent at *para* position of the benzene ring in **1d**–**g**, the selectivity of *E* and *Z* and the isolated yield of the *E* isomer seemed to be good. In the case of unsymmetrical diarylacetylene, 1-(*tert*-butyl)-4-((4-fluorophenyl)ethynyl)benzene (**1h**) was tested and the corresponding ***E*-2h** was isolated and purified with MeOH. ***E*-2h** was obtained in 41% yield (Table 2, entry 7).

**Table 2:** Scope and limitations for bromination of various diarylacetylenes **1**.

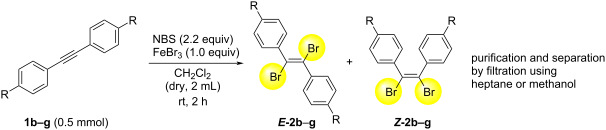			

Entry	Product	Ratio of ***E*-2**/***Z*-2**^a^	Yield (%) of ***E-2***

1^b^	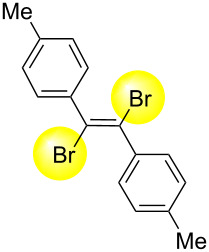 ***E*** **-2b**	68:32	<54^c^
2^b^	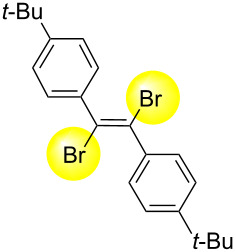 ***E*** **-2c**	57:43	45
3^b^	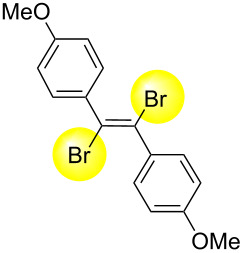 ***E*** **-2d**	82:18	75
4^b^	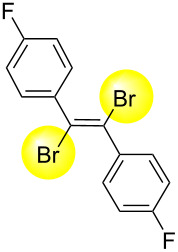 ***E*** **-2e**	89:11	56
5^d^	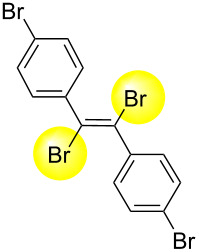 ***E*** **-2f**	89:11	78
6^b^	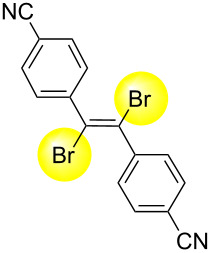 ***E*** **-2g**	82:18^e^	70
7^b^	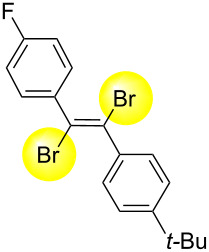 ***E*** **-2h**	78:22	41

^a^Calculated from the ratio of GC area for crude product. ^b^Isolated yield of *E*-isomer by filtration with methanol. ^c^Because a small amount of impurity after the isolation was confirmed, the chemical yield was expressed as ’’<54% yield’’. See NMR spectra in Supporting Information File 1. ^d^Isolated yield of *E*-isomer by filtration with heptane. ^e^Calculated from the ratio of ^1^H NMR for crude product.

**Scheme 2:**
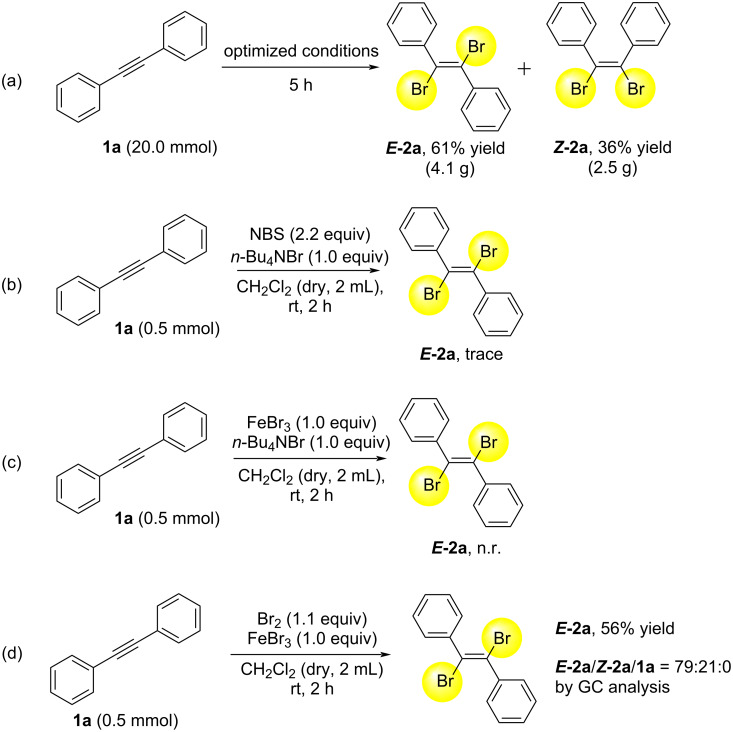
Gram-scale synthesis of (a), and control experiments of (b), (c) and (d). n.r. = no reaction.

A gram-scale synthesis of ***E*-2a** and ***Z*-2a** from **1a** was performed (Scheme 2a). **1a** (20.0 mmol) was allowed to react under optimized conditions for 5 hours. The purification and separation by using heptane from the crude product afforded ***E*-2a** (4.1 g, 12.2 mmol, 61% yield) and ***Z*-2a** (2.5 g, 7.2 mmol, 36% yield), indicating that purification and separation by heptane make scale-up very easy. The *E*-selectivity was dropped in gram-scale synthesis, although the reason is unclear at present.

For the understanding of the reaction mechanism, some control experiments were carried out. The reaction of **1a** with NBS and *n*-Bu_4_NBr without FeBr_3_ gave trace amount of ***E*-2a** (Scheme 2b). In addition, the reaction of **1a** with FeBr_3_ and *n*-Bu_4_NBr without NBS showed no reaction (Scheme 2c). Instead of NBS, Br_2_ (1.1 equiv) was reacted with **1a** and FeBr_3_ (1.0 equiv) to give ***E*-2a** in 56% yield, with a ratio of ***E*-2a**/***Z*-2a**/**1a** = 79:21:0. These results indicate that the combination of NBS and FeBr_3_ might be important and in situ Br_2_ might be generated and formed. FeBr_3_ might react with Br_2_ to form [FeBr_4_]^−^ and "Br^+^", which can react with **1a**.

**Scheme 3:**
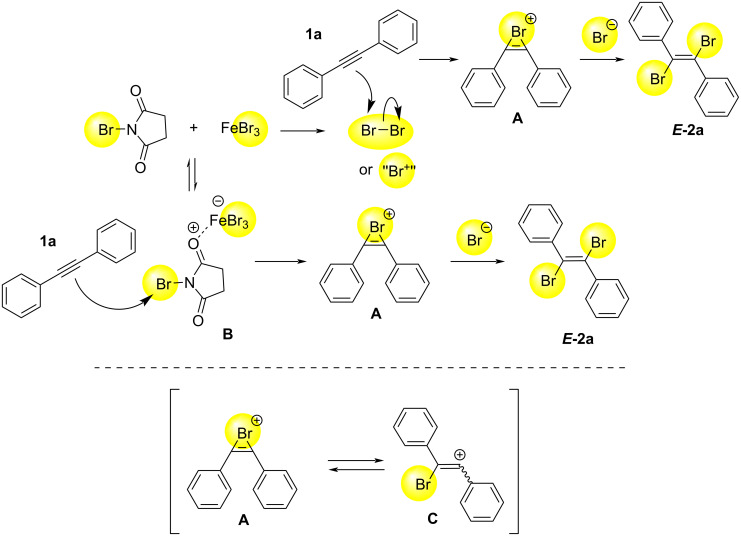
Plausible reaction mechanism of the present reaction.

As for the reaction mechanism, the plausible pathway might be the following (Scheme 3). The reaction of NBS and FeBr_3_ might generate Br_2_ or "Br^+^", which can react with **1a** to form intermediate **A**. **A** reacts with Br^−^ to give ***E*-2a**. Another possibility is the reaction of NBS and FeBr_3_ gives coordinated intermediate **B**, which can react with **1a** to give **A**, leading to the formation of ***E*-2a** [21–22]. Both pathways may be mixed together. One reason why *E* and *Z* isomers are formed together might be due to the equilibrium between **A** and **C**, although there are many unknown points.

## Conclusion

In summary, we have developed a simple and selective procedure of the synthesis of (*E*)-1,2-dibromo-1,2-diphenylethylene from diphenylacetylene by using NBS and FeBr_3_ in CH_2_Cl_2_. In addition, heptane played an important and critical role for purification and separation of the crude product. Scope and limitations for bromination of various diarylacetylenes together with the gram-scale synthesis and control experiments were also investigated. These results and findings can contribute to process chemistry for the synthesis of (*E*)-1,2-dibromo-1,2-diphenylethylene and their derivatives. Further synthetic investigations are currently underway in our laboratory.

## Experimental

The experimental procedures and characterization data (Table 1, entry 1). The glass flask was dried and heated by a heating gun. After cooled to room temperature, the flask was filled with N_2_. FeBr_3_ (154.2 mg, 0.52 mmol) and NBS (*N*-bromosuccinimide, 196.0 mg, 1.10 mmol) were added to the glass flask. Then, CH_2_Cl_2_ (dry, 2.0 mL) and 1,2-diphenylacetylene (**1a**, 89.4 mg, 0.502 mmol) were added and the mixture was stirred at room temperature for 2 hours. A 10% aqueous solution of Na_2_S_2_O_3_ (20 mL) was added to stop the reaction. The mixture was extracted with CH_2_Cl_2_ (20 mL × 1), and separated. The aqueous phase was extracted with CH_2_Cl_2_ (20 mL × 2). The combined organic phase was washed with H_2_O (20 mL) and brine (20 mL), and dried over Na_2_SO_4_. After this, filtration and concentration were performed; the organic phase was passed through a short column of silica gel using CH_2_Cl_2_ (100 mL) to remove inorganic materials and others; then it was concentrated under reduced pressure to give the crude product. This crude product was purified three times with heptane to obtain (*E*)-1,2-dibromo-1,2-diphenylethylene (***E*-2a**, 132.6 mg, 0.392 mmol, 78% yield) of high-purity.

**(*E*)-1,2-Dibromo-1,2-diphenylethylene (*E*-2a)** [4]: White solid. ^1^H NMR (400 MHz, CDCl_3_) δ 7.34–7.46 (m, 6H), 7.50–7.57 (m, 4H) ppm; ^13^C NMR (100 MHz CDCl_3_) δ 118.0, 128.4, 128.9, 129.1, 140.7 ppm; HRMS (ESI) *m*/*z*: [M + Na]^+^ calcd for C_14_H_10_Br_2_Na, 358.9041; found, 358.9028.

**(*Z*)-1,2-Dibromo-1,2-diphenylethylene (*Z*-2a)** [7]: Yellow solid. ^1^H NMR (300 MHz, CDCl_3_) δ 7.10–7.22 (m, 10H) ppm; ^13^C NMR (75 MHz, CDCl_3_) δ 125.7, 128.0, 128.3, 129.8, 139.4 ppm; HRMS (ESI) *m*/*z*: [M + Na]^+^ calcd for C_14_H_11_Br_2_, 336.9222; found, 336.9215.

## Supporting Information

File 1 General remarks, preparation of substrates, experimental procedure, characterization data of compounds, and copies of ^1^H and ^13^C NMR spectra.

## Data Availability

All data that supports the findings of this paper is available in the published article and/or the supporting information to this article.

## References

[1] Saikia I, Borah A J, Phukan P (2016). Chem Rev.

[2] Gombos L G, Waldvogel S R (2022). Sustainable Chem.

[3] Espenson J H, Zhu Z, Zauche T H (1999). J Org Chem.

[4] Schuh K, Glorius F (2007). Synthesis.

[5] Adimurthy S, Ghosh S, Patoliya P U, Ramachandraiah G, Agrawal M, Gandhi M R, Upadhyay S C, Ghosh P K, Ranu B C (2008). Green Chem.

[6] Ma K, Li S, Weiss R G (2008). Org Lett.

[7] Yao M-L, Kabalka G W, Blevins D W, Reddy M S, Yong L (2012). Tetrahedron.

[8] Sohmiya H, Kimura T, Fujita M, Ando T (1998). Tetrahedron.

[9] Windmon N, Dragojlovic V (2008). Tetrahedron Lett.

[10] Cristiano R, Ma K, Pottanat G, Weiss R G (2009). J Org Chem.

[11] Dağalan Z, Koçak R, Daştan A, Nişancı B (2022). Org Lett.

[12] Podgoršek A, Eissen M, Fleckenstein J, Stavber S, Zupan M, Iskra J (2009). Green Chem.

[13] Okumura H, Prakoso N I, Morozumi T, Umezawa T (2024). Org Lett.

[14] Muathen H A (2004). Synth Commun.

[15] Cho E, Jayaraman A, Lee J, Ko K C, Lee S (2019). Adv Synth Catal.

[16] Hamasaki K, Tomiyama R, Yoneyama S, Xu P, Matsumoto K (2024). Electrochemistry.

[17] Suzuki H, Togo A, Kikuzawa J, Miyamoto K, Marumoto S, Kuwabara A, Kobayashi M, Matsumoto K (2024). Chem Lett.

[18] Fujiki Y, Suzuki H, Kikuzawa J, Nishiwaki K, Kawashita N, Matsuoka J, Nakamura A, Maegawa T, Kuwabara A, Kobayashi M (2024). Chem Lett.

[19] Miyamoto K, Kikuzawa J, Togo A, Fujiki Y, Kawashita N, Matsumoto K (2025). Bull Chem Soc Jpn.

[20] Kong Y, Cao T, Zhu S (2021). Chin J Chem.

[21] Zheng Y F, Yu J, Yan G B, Li X, Luo S (2011). Chin Chem Lett.

[22] Catano B, Lee J, Kim C, Farrell D, Petersen J L, Xing Y (2015). Tetrahedron Lett.

