# Supplementary material for: Synthetic study of vic-bromination of diarylacetylenes, easy purification and separation
Source: Beilstein J Org Chem. 2026 May 22;22:795–802. doi: 10.3762/bjoc.22.61 (PMC13202478; doi:10.3762/bjoc.22.61)

BEILSTEIN JOURNAL OF ORGANIC CHEMISTRY

Supporting Information

for

Synthetic study of *vic*-bromination of diarylacetylenes, easy purification and separation

Akane Togo, Hiyono Suzuki, Yuto Akai, Makoto Matsumoto, Yoshinori Suzuma, Hidehiko Kodama and Kouichi Matsumoto

Beilstein J. Org. Chem. **2026,** *22,* 795–802. doi:10.3762/bjoc.22.61

General remarks, preparation of substrates, experimental procedure, characterization data of compounds, and copies of ^{1}H and ^{13}C NMR spectra

License and Terms: This is a supporting information file under the terms of the Creative Commons Attribution License (https://creativecommons.org/licenses/by/4.0). Please note that the reuse, redistribution and reproduction in particular requires that the author(s) and source are credited and that individual graphics may be subject to special legal provisions.
The license is subject to the *Beilstein Journal of Organic Chemistry* terms and conditions: (https://www.beilstein-journals.org/bjoc/terms)

1. General remarks

Varian MERCURY 300 (^{1}H NMR 300 MHz, ^{13}C NMR 75 MHz), JEOL JNM-ECS 400 (^{1}H NMR 400 MHz, ^{13}C NMR 100 MHz), and BRUKER AVANCE NEO 400 (^{1}H NMR 400 MHz, ^{13}C NMR 100 MHz) were used for the analysis of organic compounds. $CDCl_3$ was used, and the chemical shift of ^{1}H NMR was based on 0.00 ppm of Me_4Si (tetramethylsilane), and the chemical shift of ^{13}C NMR was based on 77.0 ppm. Thermo Fisher Scientific Exactive Plus was used for the analysis of high resolution mass spectrometry. Shimadzu DGU-20A5R (degasser), LC-20AD (pump), SIL-20ACHT (autosampler), SPD-M20A (lamp), and CTO-20AC (column oven) equipped with Waters Corporation XBridge C18 were used for the analysis of HPLC, in which MeCN and 20 mM HCO_2NH_4 aqueous solution were used as solvents in HPLC. Shimadzu GC-2014 was used for GC analysis, in which the following condition was adopted. (Oven temp: 130 °C to 250 °C at 10 °C/min, hold for 15 min, or Oven temp: 130 °C to 250 °C at 6 °C/min, hold for 10 min). Agilent 7890A Mass Selective Detector, equipped with Agilent 5975C inert XL MSD with Triple Axis was used for the analysis of GC-MS (EI). For some reactions, Biotage Isolera LS and Isolera One flash automated purification systems were used. Precoated silica gel F254 plates (thickness 0.25 mm) and silica gel 60 F254 (TLC aluminum sheets) from Merck were used for TLC analysis. Silica gel was used from Kanto Chemical Co., Ltd. (Silica Gel N, spherical, neutral, 40–100 μm), Fuji Silysia Chemical Ltd. (BW-200), and Biotage Inc. (Sfär Silica High Capacity Duo 20 μm). Two types of dry dichloromethane were used. One was purchased from Kanto Chemical Co., Ltd. and used. Second was prepared by the laboratory according to the literature, after CH_2Cl_2 containing MeOH as a stabilizer was purchased.[1]

2. Materials

Unless otherwise mentioned, all reagents and solvents were purchased and used without further purification. Diarylacetylenes except **1a** and **1f** were synthesized as follows.

Typical procedure of synthesis of 1,2-bis(4-methoxyphenyl)ethyne (1d)

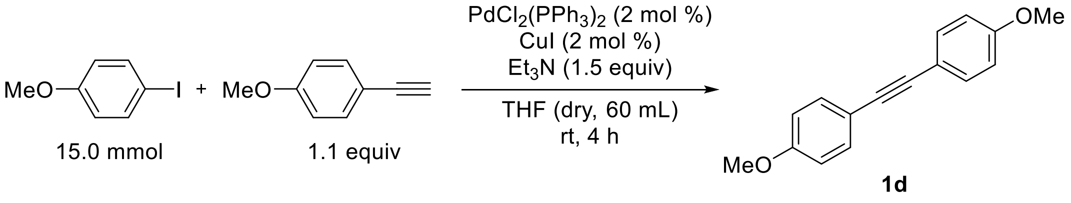

Glass flask was dried and heated by heating gun, under vacuum. After cooled to room temperature, N_2 was placed. CuI (57.5 mg, 0.30 mmol), $PdCl_2(PPh_3)_2$ (211.3 mg, 0.30 mmol), THF (dry, 60 mL), 1-iodo-4-methoxybenzene (3.51 g, 15.0 mmol), Et_3N (3.10 mL, d = 0.73 g/mL, ca. 2.26 g, ca. 22.3 mmol), and 1-ethynyl-4-methoxybenzene (2.18 g, 16.5 mmol) were added, and the solution was stirred

at room temperature for 4 h. The reaction was quenched by the addition of H_2O (30 mL). AcOEt (20 mL) was added to the mixture, and it was separated. The aqueous phase was extracted by AcOEt (20 mL × 2). The combined organic phase was washed by brine (20 mL), and dried over Na_2SO_4. The purification was conducted by column chromatography of silica gel to give 1,2-bis(4-methoxyphenyl)ethyne (**1d**, 2.59 g, 10.9 mmol, 73% yield). Other materials such as **1b**, **1c**, **1e, 1g,** and **1h** were also synthesized by using the similar procedure. **1a** and **1f** were purchased and used without further purification.

1,2-Di-*p*-tolylethyne (1b)[2]

GC analysis of isolated compound indicated 86% of the purity.

40% yield. Yellow solid. TLC R_f = 0.56 (hexane/AcOEt = 20:1); ^{1}H NMR (400 MHz, $CDCl_3$): δ 2.36 (s, 6H), 7.14 (d, J = 7.6 Hz, 4H), 7.41 (d, J = 8.4 Hz, 4H) ppm; ^{13}C NMR (100 MHz, $CDCl_3$): δ 21.5, 88.9, 120.4, 129.1, 131.4, 138.2 ppm; HRMS (ESI) calculated for $C_{16}H_{15}$ ($[M+H]^+$): 207.1168; found: 207.1165.

1,2-Bis(4-(*tert*-butyl)phenyl)ethyne (1c)[3]

GC analysis of isolated compound indicated 97% of the purity. In ^{13}C NMR, 132.2 ppm derived from impurity was observed. The analysis of 131.89 ppm in ^{13}C NMR of the reported literature of ref. 3a seems to be incorrect.

66% yield. Light yellow solid. TLC R_f = 0.58 (hexane/AcOEt = 20:1); ^{1}H NMR (400 MHz, $CDCl_3$): δ 1.32 (s, 18H), 7.33-7.38 (m, 4H), 7.43-7.48 (m, 4H) ppm; ^{13}C NMR (100 MHz, $CDCl_3$): δ 31.2, 34.8, 88.8, 120.4, 125.3, 131.3, 151.3 ppm; HRMS (ESI) calculated for $C_{22}H_{27}$ ($[M+H]^+$): 291.2107; found: 291.2104.

1,2-Bis(4-methoxyphenyl)ethyne (1d)[2a,4,5]

73% yield. Light orange solid. TLC R_f = 0.58 (hexane/CH_2Cl_2 = 1:1); ^{1}H NMR (400MHz, $CDCl_3$): δ 3.82 (s, 6H), 6.87 (d, J = 8.8 Hz, 4H), 7.45 (d, J = 8.8 Hz, 4H) ppm; ^{13}C NMR (100 MHz, $CDCl_3$): δ 55.3, 87.9, 113.9, 115.7, 132.8, 159.4 ppm; HRMS (ESI) calculated for $C_{16}H_{15}O_2$ ($[M+H]^+$): 239.1067; found: 239.1061.

1,2-Bis(4-fluorophenyl)ethyne (1e)[2,3a]

GC analysis of isolated compound indicated 88% of the purity.

84% yield. Yellow solid. TLC R_f = 0.60 (hexane/AcOEt = 20:1); ^{1}H NMR (400 MHz, $CDCl_3$): δ 6.98-7.09 (m, 4H), 7.45-7.55 (m, 4H) ppm; ^{13}C NMR (100 MHz, $CDCl_3$): δ 87.9, 115.7 (d, J = 21.9 Hz), 119.2 (d, J = 3.8 Hz), 133.4 (d, J = 8.6 Hz), 162.5 (d, J = 248.9 Hz) ppm; HRMS (ESI) calculated for $C_{14}H_9F_2$ ($[M+H]^+$): 215.0667; found: 215.0673.

4,4'-(Ethyne-1,2-diyl)benzonitrile (1g)[4]

64% yield. Light yellow solid. TLC R_f = 0.15 (hexane/CH_2Cl_2 = 1:1); ^{1}H NMR (400 MHz, $CDCl_3$): δ 7.61-7.70 (m, 8H) ppm; ^{13}C NMR (100 MHz, $CDCl_3$): δ 91.5, 112.4, 118.2, 127.0, 132.2, 132.3 ppm; HRMS (ESI) calculated for $C_{16}H_8N_2Na$ ($[M+Na]^+$): 251.0580; found: 251.0573.

1-(*tert*-Butyl)-4-((4-fluorophenyl)ethynyl)benzene (1h)[5]

81% yield. White solid. TLC R_f = 0.73 (hexane/AcOEt = 20:1) ^{1}H NMR (400 MHz, $CDCl_3$): δ 1.33 (s, 9H), 7.00-7.07 (m, 2H), 7.34-7.39 (m, 2H), 7.43-7.53 (m, 4H) ppm; ^{13}C NMR (100 MHz, $CDCl_3$): δ 31.2, 34.8, 87.6, 89.2, 115.6 (d, J = 22.0 Hz), 119.6 (d, J = 2.9 Hz), 120.0, 125.4, 131.3, 133.4 (d, J = 8.6 Hz), 151.6, 162.4 (d, J = 247.9 Hz) ppm; HRMS (ESI) calculated for $C_{18}H_{18}F$ ($[M+H]^+$): 253.1387; found: 253.1394.

3. Typical procedure for the reaction of diphenylacetylene (1a), NBS, and $FeBr_3$ to (*E*)-1,2-dibromo-1,2-diphenylethylene (*E*-2a) (Table 1, entry 1)

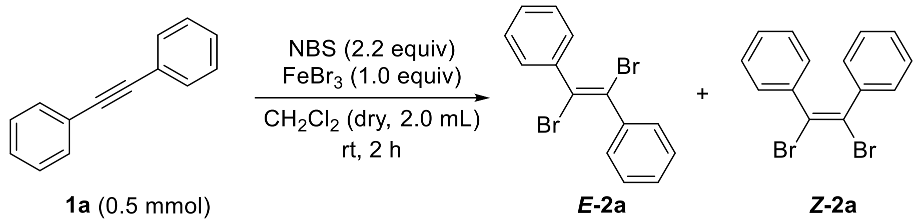

Glass flask was dried and heated by heating gun, under vacuum. After cooled to room temperature, N_2 was placed. $FeBr_3$ (154.2 mg, 0.52 mmol) and NBS (*N*-bromosuccinimide, 196.0 mg, 1.10 mmol) were added to the glass flask. Then, CH_2Cl_2 (dry, 2.0 mL) was added. 1,2-Diphenylacetylene (**1a**, 89.4 mg, 0.502 mmol) was added and the mixture was stirred at room temperature for 2 h. The 10% aqueous solution of $Na_2S_2O_3$ (20 mL) was added and the reaction was stopped. The mixture was extracted by CH_2Cl_2 (20 mL × 1), and separated. The aqueous phase was extracted by CH_2Cl_2 (20 mL × 2). The combined organic phase was washed by H_2O (20 mL) and brine (20 mL), and dried over Na_2SO_4. Then, filtration and concentration were performed, and organic material was passed through a short column of silica gel using CH_2Cl_2 (100 mL) to remove inorganic materials and others, which was concentrated under reduced pressure to give crude product.

This crude product was purified (filtered) three times with heptane to obtain (*E*)-1,2-dibromo-1,2-diphenylethylene (***E*-2a**, 132.6 mg, 0.392 mmol, 78% yield) of high-purity. Purification using heptane involved separating the solid and filtrate, concentrating the filtrate, and then filtering it with new heptane. This process was repeated. Three solids were collected and combined into one.

Notification: Since *Z* isomer generally dissolved, it was separated by filtration from *E* and *Z* isomers. Because of this reason, its purity of *Z* isomers was not high in Table 1 and Table 2. The following NMR spectra also showed that the *Z* isomers such as **2a**, **2b**, **2c**, **2d**, **2e**, **2f**, **2g** and **2h** had low purity. In Table 1 and Table 2, isolated yields meant *E* isomer, and yields of *Z* isomer were not included.

(*E*)-1,2-Dibromo-1,2-diphenylethylene (*E*-2a)[6]

White solid. ^{1}H NMR (400 MHz, $CDCl_3$): δ 7.34-7.46 (m, 6H), 7.50-7.57 (m, 4H) ppm; ^{13}C NMR (100 MHz, $CDCl_3$): δ 118.0, 128.4, 128.9, 129.1, 140.7 ppm; HRMS (ESI) calculated for $C_{14}H_{10}Br_2Na$ ([M+Na]$^+$): 358.9041; found: 358.9028.

(*Z*)-1,2-Dibromo-1,2-diphenylethylene (*Z*-2a)[7]

Orange solid. ^{1}H NMR (300 MHz, $CDCl_3$): δ 7.10-7.22 (m, 10H) ppm; ^{13}C NMR (75 MHz, $CDCl_3$): δ 125.7, 128.0, 128.3, 129.8, 139.4 ppm; HRMS (ESI) calculated for $C_{14}H_{11}Br_2$ ([M+H]$^+$): 336.9222; found: 336.9215.

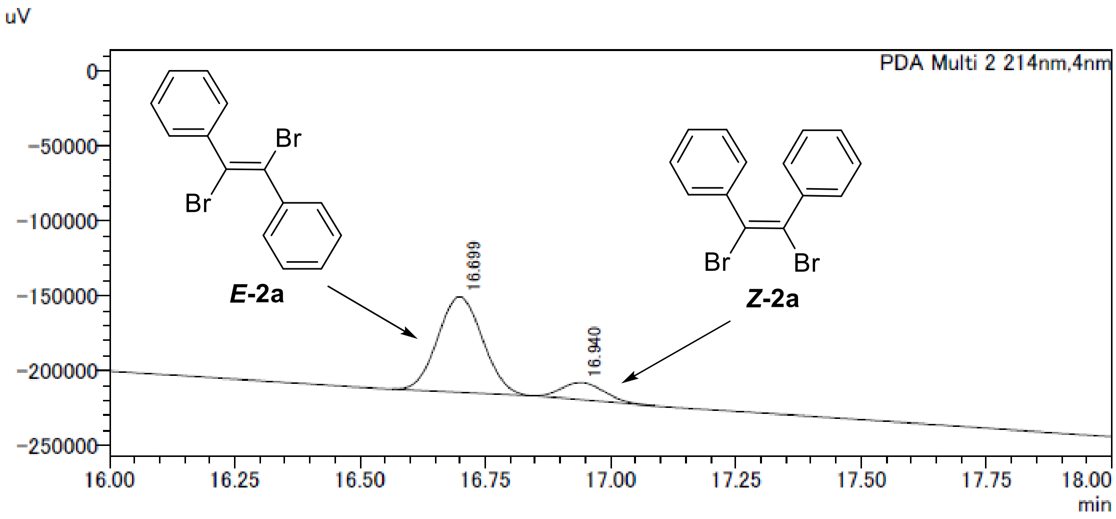

Figure S1. HPLC detection and analysis of ***E*-2a** and ***Z*-2a** at the stage of crude product (Table 1, entry 1).

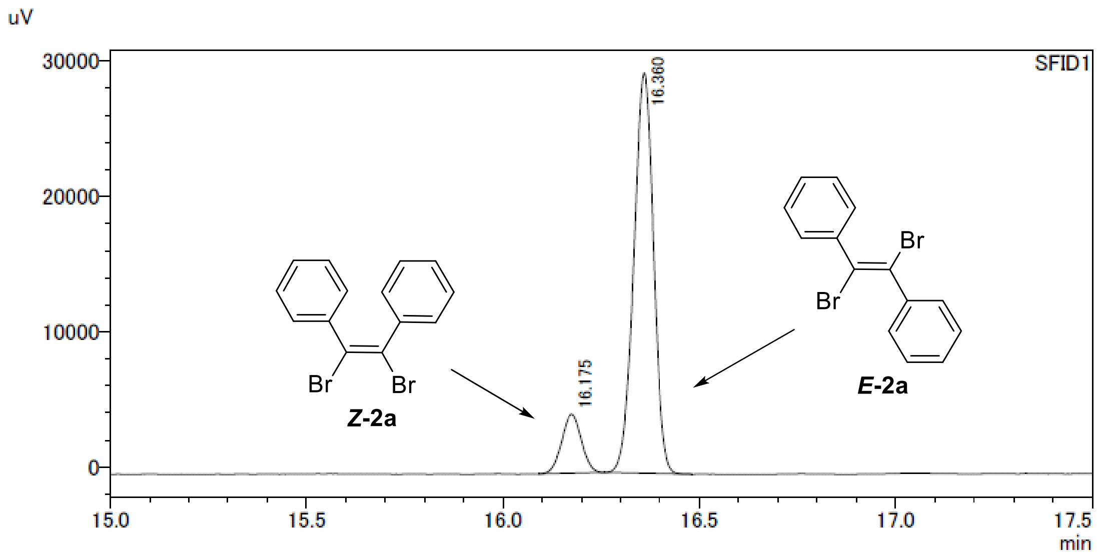

Figure S2. GC detection (FID) and analysis of ***E*-2a** and ***Z*-2a** at the stage of crude product (Table 1, entry 2).

(*E*)-1,2-Dibromo-1,2-di-*p*-tolylethylene (*E*-2b) (Table 2, entry 1)

<54% yield. Green solid. ^{1}H NMR (400 MHz, $CDCl_3$): δ 2.39 (s, 6H), 7.22 (d, *J* = 8.0 Hz, 4H), 7.42 (d, *J* = 8.4 Hz, 4H) ppm; ^{13}C NMR (100 MHz, $CDCl_3$): δ 21.4, 117.9, 128.99, 129.03, 138.0, 138.9 ppm; HRMS (ESI) calculated for $C_{16}H_{14}Br_2Na$ ([M+Na]$^+$): 386.9354; found: 386.9347.

(*Z*)-1,2-Dibromo-1,2-di-*p*-tolylethylene (*Z*-2b) (Table 2, entry 1)

Green solid. ^{1}H NMR (400 MHz, $CDCl_3$): δ 2.24 (s, 6H), 6.94 (d, *J* = 8.4 Hz, 4H), 7.08 (d, *J* = 8.4 Hz, 4H) ppm; ^{13}C NMR (100 MHz, $CDCl_3$): δ 21.2, 125.4, 128.7, 129.7, 136.6, 138.2 ppm; HRMS (ESI) calculated for $C_{16}H_{15}Br_2$ ([M+H]$^+$): 364.9535; found: 364.9531.

(*E*)-1,2-Dibromo-1,2-bis(4-(*tert*-butyl)phenyl)ethylene (*E*-2c) (Table 2, entry 2)[8]

45% yield. White solid. ^{1}H NMR (400 MHz, $CDCl_3$): δ 1.35 (s, 18H), 7.39-7.50 (m, 8H) ppm; ^{13}C NMR (100 MHz, $CDCl_3$): δ 31.2, 34.8, 117.8, 125.2, 128.8, 137.9, 151.9 ppm; HRMS (ESI) calculated for $C_{22}H_{26}Br_2Na$ ([M+Na]$^+$): 471.0293; found: 471.0277.

(*Z*)-1,2-Dibromo-1,2-bis(4-(*tert*-butyl)phenyl)ethylene (*Z*-2c) (Table 2, entry 2)

Yellow solid. ^{1}H NMR (400 MHz, $CDCl_3$): δ 1.23 (s, 18H), 7.07-7.19 (m, 8H) ppm; ^{13}C NMR (100 MHz, $CDCl_3$): δ 31.1, 34.5, 124.8, 125.2, 129.5, 136.5, 151.3 ppm; HRMS (ESI) calculated for $C_{22}H_{27}Br_2$ ([M+H]$^+$): 449.0474; found: 449.0472.

(*E*)-1,2-Dibromo-1,2-bis(4-methoxyphenyl)ethylene (*E*-2d) (Table 2, entry 3)
75% yield. White solid. ^{1}H NMR (300 MHz, $CDCl_3$): δ 3.85 (s, 6H), 6.93 (d, *J* = 8.7 Hz, 4H), 7.47 (d, *J* = 8.7 Hz, 4H) ppm; ^{13}C NMR (75 MHz, $CDCl_3$): δ 55.3, 113.6, 117.7, 130.7, 133.3, 159.7 ppm; HRMS (ESI) calculated for $C_{16}H_{14}Br_2O_2Na$ ($[M+Na]^+$): 418.9253; found: 418.9235.

(*Z*)-1,2-Dibromo-1,2-bis(4-methoxyphenyl)ethylene (*Z*-2d) (Table 2, entry 3)
Yellow solid. ^{1}H NMR (400 MHz, $CDCl_3$): δ 3.74 (s, 6H), 6.67 (d, *J* = 8.8 Hz, 4H), 7.12 (d, *J* = 8.4 Hz, 4H) ppm; ^{13}C NMR (100 MHz, $CDCl_3$): δ 55.2, 113.4, 124.9, 131.2, 132.0, 159.2 ppm; HRMS (ESI) calculated for $C_{16}H_{15}Br_2O_2$ ($[M+H]^+$): 396.9433; found: 396.9427.

(*E*)-1,2-Dibromo-1,2-bis(4-fluorophenyl)ethylene (*E*-2e) (Table 2, entry 4)
56% yield. White solid. ^{1}H NMR (400 MHz, $CDCl_3$): δ 7.07-7.15 (m, 4H), 7.47-7.54 (m, 4H) ppm; ^{13}C NMR (100 MHz, $CDCl_3$): δ 115.5 (d, *J* = 22.0 Hz), 117.5, 131.1 (d, *J* = 8.5 Hz), 136.6 (d, *J* = 2.9 Hz), 162.6 (d, *J* = 248.8 Hz) ppm; HRMS (ESI) calculated for $C_{14}H_8Br_2F_2Na$ ($[M+Na]^+$): 394.8853; found: 394.8850.

(*Z*)-1,2-Dibromo-1,2-bis(4-fluorophenyl)ethylene (*Z*-2e) (Table 2, entry 4)
Yellow solid. Because the purity of ***Z*-2e** was poor, it was difficult to analyze ***Z*-2e** by ^{1}H NMR and ^{13}C NMR. Please see following spectra of ^{1}H NMR and ^{13}C NMR.

(*E*)-1,2-Dibromo-1,2-bis(4-bromophenyl)ethylene (*E*-2f) (Table 2, entry 5) [9]
78% yield. White solid. ^{1}H NMR (400 MHz, $CDCl_3$): δ 7.39 (d, *J* = 8.4 Hz, 4H), 7.56 (d, *J* = 8.8 Hz, 4H) ppm; ^{13}C NMR (100 MHz, $CDCl_3$): δ 117.4, 123.3, 130.7, 131.7, 139.3 ppm; HRMS (ESI) calculated for $C_{14}H_9Br_4$ ($[M+H]^+$): 492.7432; found: 492.7440.

(*Z*)-1,2-Dibromo-1,2-bis(4-bromophenyl)ethylene (*Z*-2f) (Table 2, entry 5)[9]
Yellow solid. ^{1}H NMR (400 MHz, $CDCl_3$): δ 7.04 (d, *J* = 8.8 Hz, 4H), 7.31 (d, *J* = 8.8 Hz, 4H) ppm. Because the purity of ***Z*-2f** was poor, it was difficult to analyze ***Z*-2f** by ^{13}C NMR. Please see following spectrum of ^{13}C NMR.

(*E*)-4,4'-(1,2-Dibromoethene-1,2-diyl)dibenzonitrile (*E*-2g) (Table 2, entry 6) [10]

70% yield. White solid. ^{1}H NMR (400 MHz, $CDCl_3$): δ 7.64 (d, *J* = 8.0 Hz, 4H), 7.75 (d, *J* = 8.8 Hz, 4H) ppm; ^{13}C NMR (100 MHz, $CDCl_3$): δ 113.1, 117.4, 118.1, 129.8, 132.4, 144.2 ppm; HRMS (ESI) calculated for $C_{16}H_8Br_2N_2Na$ ($[M+Na]^+$): 408.8946; found: 408.8937.

(*Z*)-4,4'-(1,2-Dibromoethene-1,2-diyl)dibenzonitrile (*Z*-2g) (Table 2, entry 6)

Yellow solid. ^{1}H NMR (400 MHz, $CDCl_3$): δ 7.28 (d, *J* = 8.8 Hz, 4H), 7.49 (d, *J* = 8.8 Hz, 4H) ppm; ^{13}C NMR (100 MHz, $CDCl_3$): δ 112.7, 117.8, 125.4, 130.3, 132.2, 143.1 ppm; HRMS (ESI) calculated for $C_{16}H_8Br_2N_2Na$ ($[M+Na]^+$): 408.8946; found: 408.8939.

(*E*)-1-(*tert*-Butyl)-4-(1,2-dibromo-2-(4-fluorophenyl)vinyl)benzene (***E*-2h**) (Table 2, entry 7)

41% yield. White solid. ^{1}H NMR (400 MHz, $CDCl_3$): δ 1.35 (s, 9H), 7.07-7.14 (m, 2H), 7.40-7.48 (m, 4H), 7.48-7.55 (m, 2H) ppm; ^{13}C NMR (100 MHz, $CDCl_3$): δ 31.2, 34.8, 115.4 (d, *J* = 22.0 Hz), 116.4, 119.0, 125.3, 128.8, 131.2 (d, *J* = 8.6 Hz), 137.0 (d, *J* = 3.8 Hz), 137.5, 152.1, 162.5 (d, *J* = 247.9 Hz) ppm; HRMS (ESI) calculated for $C_{18}H_{17}Br_2FNa$ ($[M+Na]^+$): 432.9573; found: 432.9567.

(*Z*)-1-(*tert*-Butyl)-4-(1,2-dibromo-2-(4-fluorophenyl)vinyl)benzene (***Z*-2h**) (Table 2, entry 7)

Yellow solid. ^{1}H NMR (400 MHz, $CDCl_3$, selected peaks): δ 1.23 (s, 9H), 6.78-6.85 (m, 2H), 7.13-7.19 (m, 4H) ppm. Because the purity of ***Z*-2h** was poor, it was difficult to analyze ***Z*-2h** by ^{13}C NMR. Please see following spectrum of ^{13}C NMR.

4. Gram scale synthesis of *E*-2a and *Z*-2a (Scheme 2 (a))

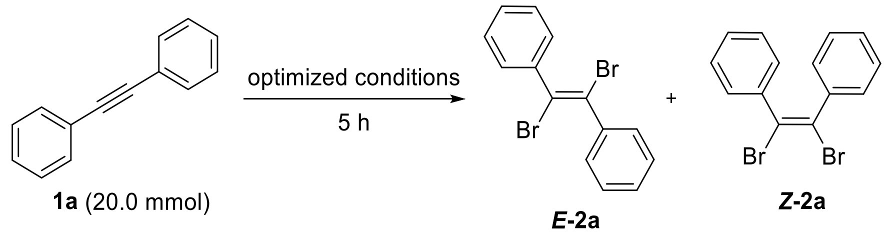

Glass flask was dried and heated by heating gun, under vacuum. After cooled to room temperature, N_2 was placed. $FeBr_3$ (5.90 g, 20.0 mmol) and NBS (*N*-bromosuccinimide, 7.85 g, 44.1 mmol) were added to the glass flask. Then, CH_2Cl_2 (dry, 80.0 mL) was added. 1,2-Diphenylacetylene (**1a**, 3.56 g, 20.0 mmol) was added and the mixture was stirred at room temperature for 5 h. The 10% aqueous solution of $Na_2S_2O_3$ (200 mL) was added and the reaction was stopped. The mixture was extracted by CH_2Cl_2 (50 mL × 1), and separated. The aqueous phase was extracted by CH_2Cl_2 (50 mL × 2). The combined organic phase was washed by H_2O (100 mL × 2) and brine (100 mL), and dried over Na_2SO_4. Then, filtration and concentration were performed, and organic material was passed through a short

column of silica gel using CH_2Cl_2 (800 mL) to remove inorganic material and others, which was concentrated under reduced pressure to give crude product.

This crude product was purified (filtered) two times with heptane (100 mL × 2) to obtain high-purity (*E*)-1,2-dibromo-1,2-diphenylethylene (***E*-2a**, 4.11 g, 12.2 mmol, 61% yield) and (*Z*)-1,2-dibromo-1,2-diphenylethylene (***Z*-2a**, 2.45 g, 7.2 mmol, 36% yield), respectively. In this case, the purity of ***Z*-2a** as well as ***E*-2a** were high by GC analysis. See NMR spectra, below. Purification using heptane involved separating the solid and filtrate, concentrating the filtrate, and then filtering it with new heptane. Two solids were collected and combined into one.

(*E*)-1,2-Dibromo-1,2-diphenylethylene (*E*-2a)[6]

61% yield. White solid. ^{1}H NMR (400 MHz, $CDCl_3$): δ 7.34-7.46 (m, 6H), 7.50-7.56 (m, 4H) ppm; ^{13}C NMR (100 MHz, $CDCl_3$): δ 118.0, 128.4, 128.9, 129.0, 140.7 ppm; HRMS (ESI) calculated for $C_{14}H_{10}Br_2Na$ ($[M+Na]^+$): 358.9041; found: 358.9032.

(*Z*)-1,2-Dibromo-1,2-diphenylethylene (*Z*-2a)[7]

36% yield. Yellow solid. ^{1}H NMR (300 MHz, $CDCl_3$): δ 7.10-7.22 (m, 10H) ppm; ^{13}C NMR (75 MHz, $CDCl_3$): δ 125.7, 128.0, 128.3, 129.8, 139.4 ppm; HRMS (ESI) calculated for $C_{14}H_{11}Br_2$ ($[M+H]^+$): 336.9222; found: 336.9215.

References

1. Ashikari, Y.; Nokami, T.; Yoshida, J. *J. Am. Chem. Soc.* **2011**, *133*, 11840.
2. (a) Li, X.; Yang, F.; Wu, Y. *RSC Adv.* **2014**, *4*, 13738. (b) Holzschneider, K.; Häring, A. P.; Kirsch, S. F. *Eur. J. Org. Chem.* **2019**, 2824.
3. (a) Peng, J-B.; Wu, P-F.; Spannenberg, A.; Wu, X-F. *Chem. Eur. J.* **2019**, *25*, 8696. (b) Herwig, P. T.; Enkelmann, V.; Schmelz, O.; Müllen, K. *Chem. Eur. J.* **2000**, *6*, 1834.
4. Wierzbicka, M.; Bylinska, I.; Czaplewski, C.; Wiczk, W. *RSC Adv.* **2015**, *5,* 29294.
5. Sahu, S. K.; Choudhury, P.; Behera, P. K.; Bisoyi, T.; Sahu, R. R.; Bisoyi, A.; Gorantla, K. R.; Mallik, B. S.; Mohapatra, M.; Rout, L. *New J. Chem.* **2022**, *46*, 1650.
6. (a) Schuh, K.; Glorius, F. *Synthesis* **2007**, *15*, 2297. (b) Podgoršek, A.; Eissen, M.; Fleckenstein, J.; Stavber, S.; Zupan, M.; Iskra, J. *Green. Chem.* **2009**, *11*, 120.
7. Yao, M-L.; Kabalka, G-W.; Blevins, D-W.; Reddy, M-S.; Yong, L. *Tetrahedron* **2012**, *68*, 3738.
8. Mataka, S.; Liu, G-B.; Torii, A.; Tashiro, M. *Bull. Chem. Soc. Jpn.* **1994**, *67*, 2336.
9. Mataka, S.; Liu, G-B.; Sawada, T.; Kurisu, M.; Tashiro, M. *Bull. Chem. Soc. Jpn*. **1994**, *67*, 1113.
10. Cho, E.; Jayaraman, A.; Lee, J.; Ko, K-C.; Lee, S. *Adv. Synth. Catal.* **2019**, *361*, 1846.

^{1}H NMR (400 MHz, $CDCl_3$) of **1b**

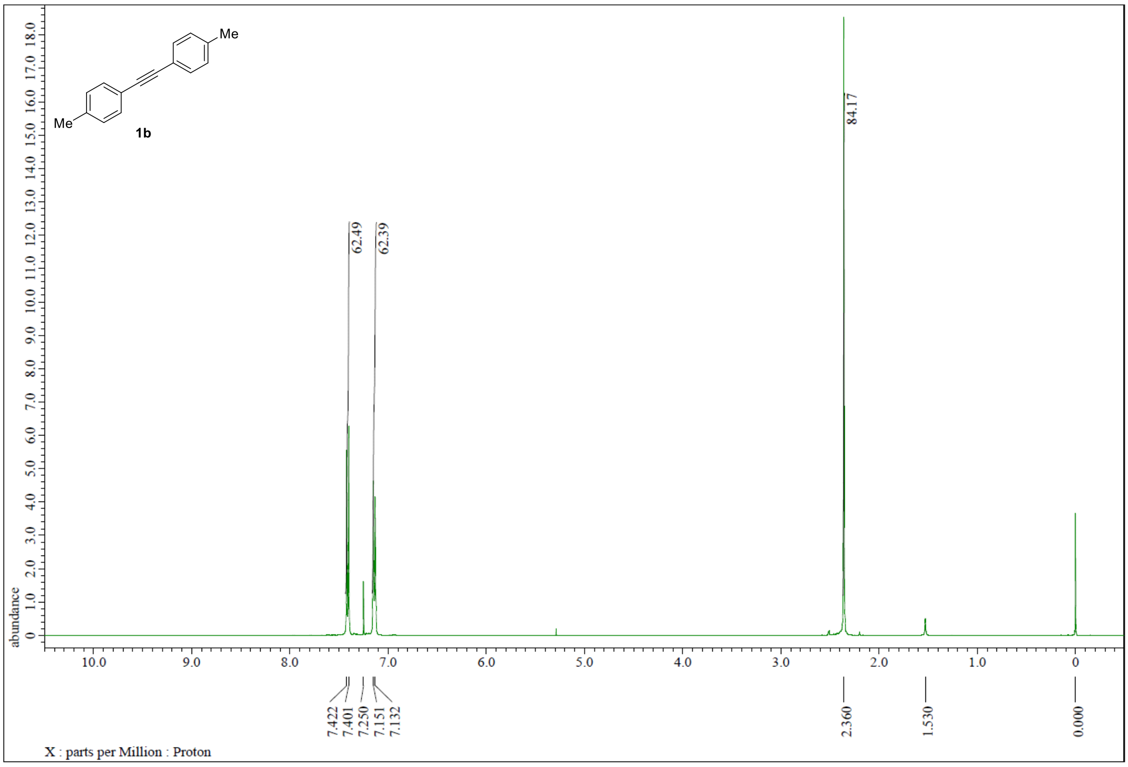

^{13}C NMR (100 MHz, $CDCl_3$) of **1b**. A small amount of impurity was confirmed.

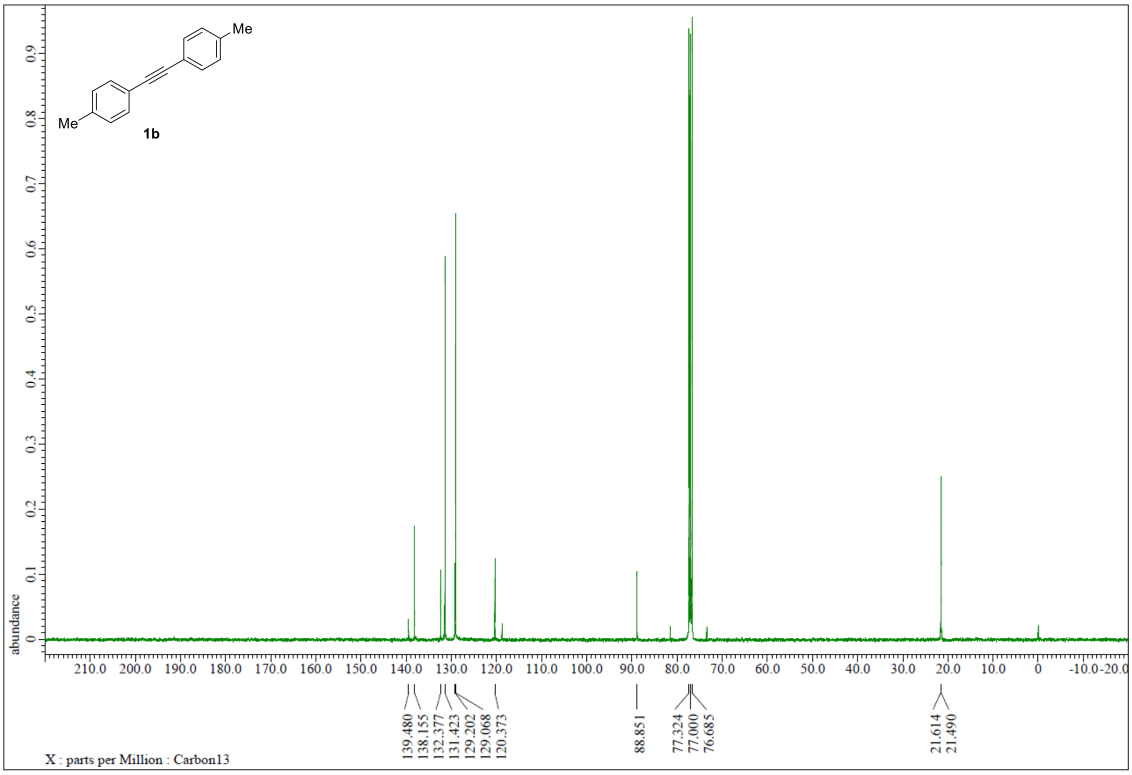

^{1}H NMR (400 MHz, $CDCl_3$) of **1c**

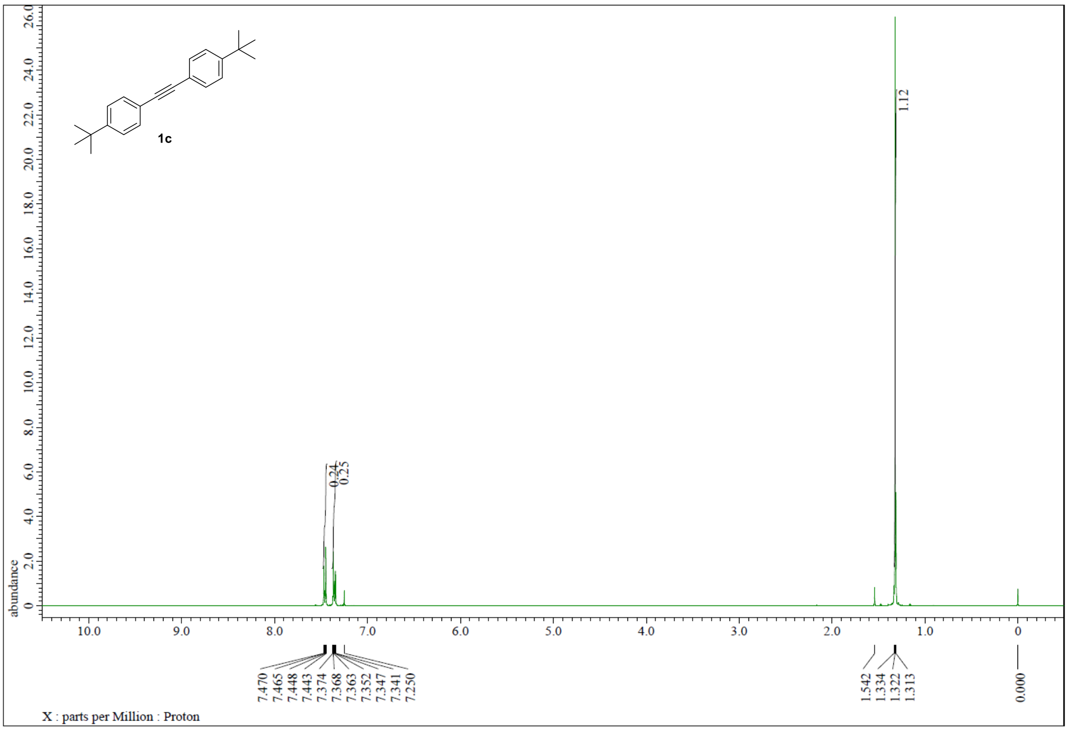

^{13}C NMR (100 MHz, $CDCl_3$) of **1c**. In ^{13}C NMR spectrum, 132.2 ppm derived from impurity was observed. Other signals of impurity were also confirmed.

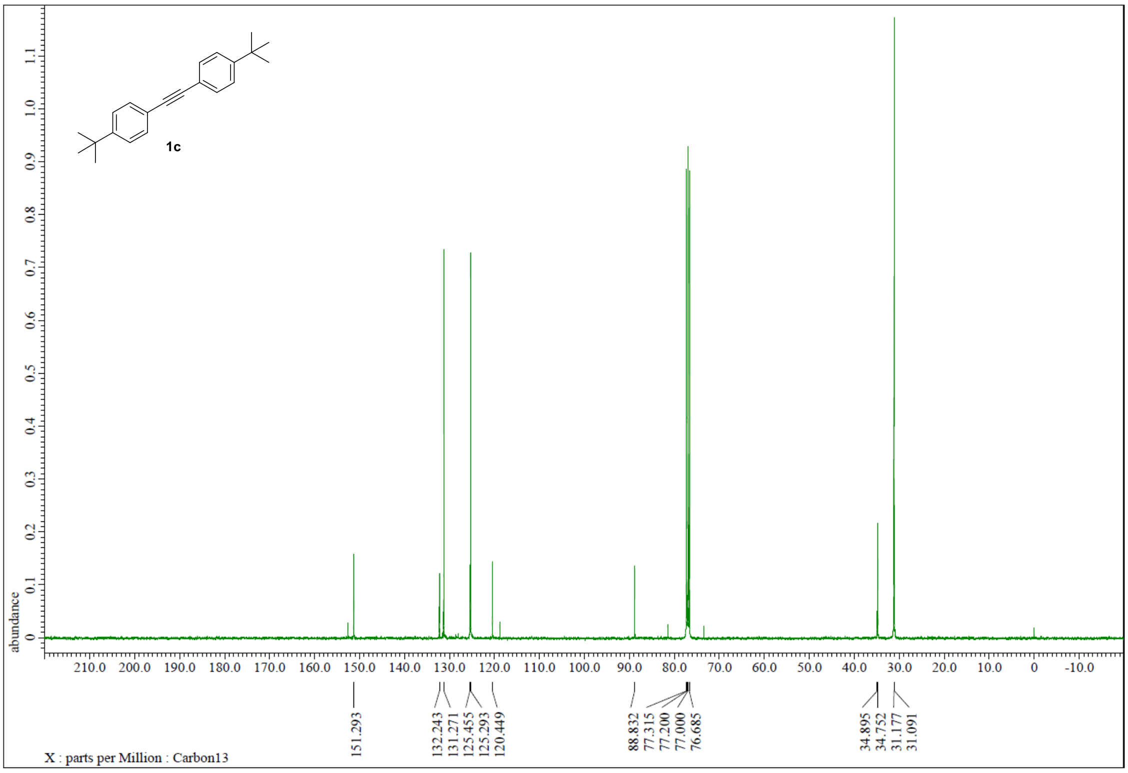

^{1}H NMR (400 MHz, $CDCl_3$) of **1d**

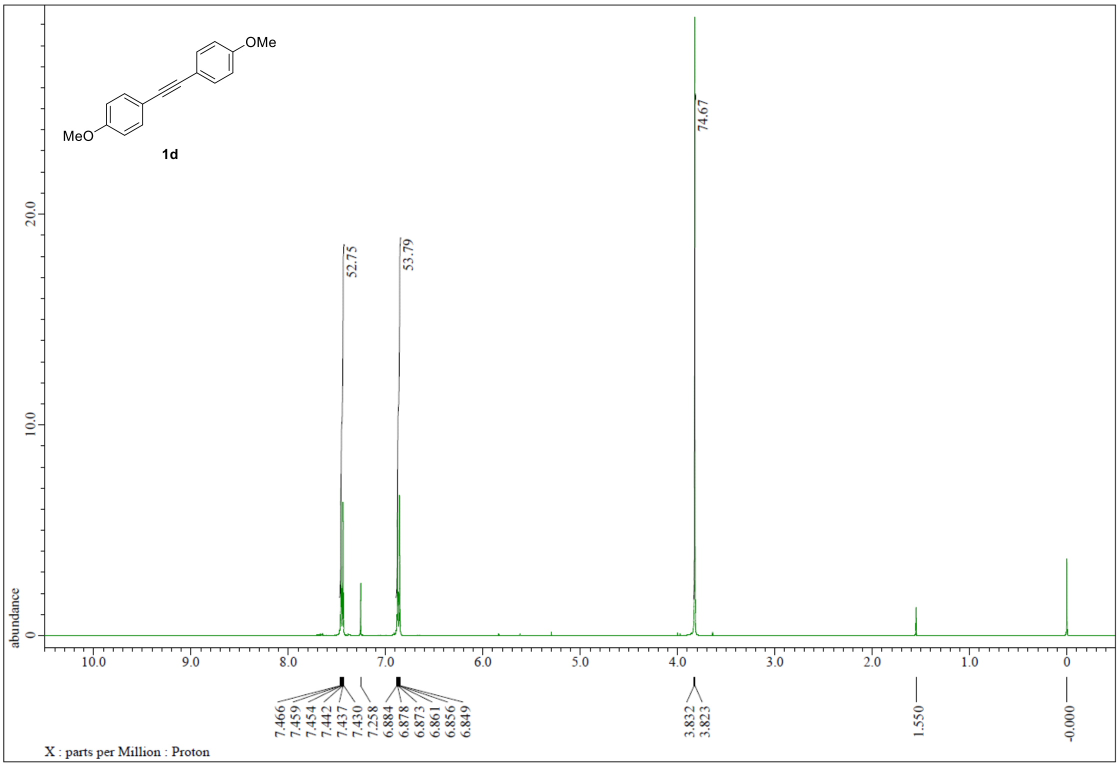

^{13}C NMR (100 MHz, $CDCl_3$) of **1d**

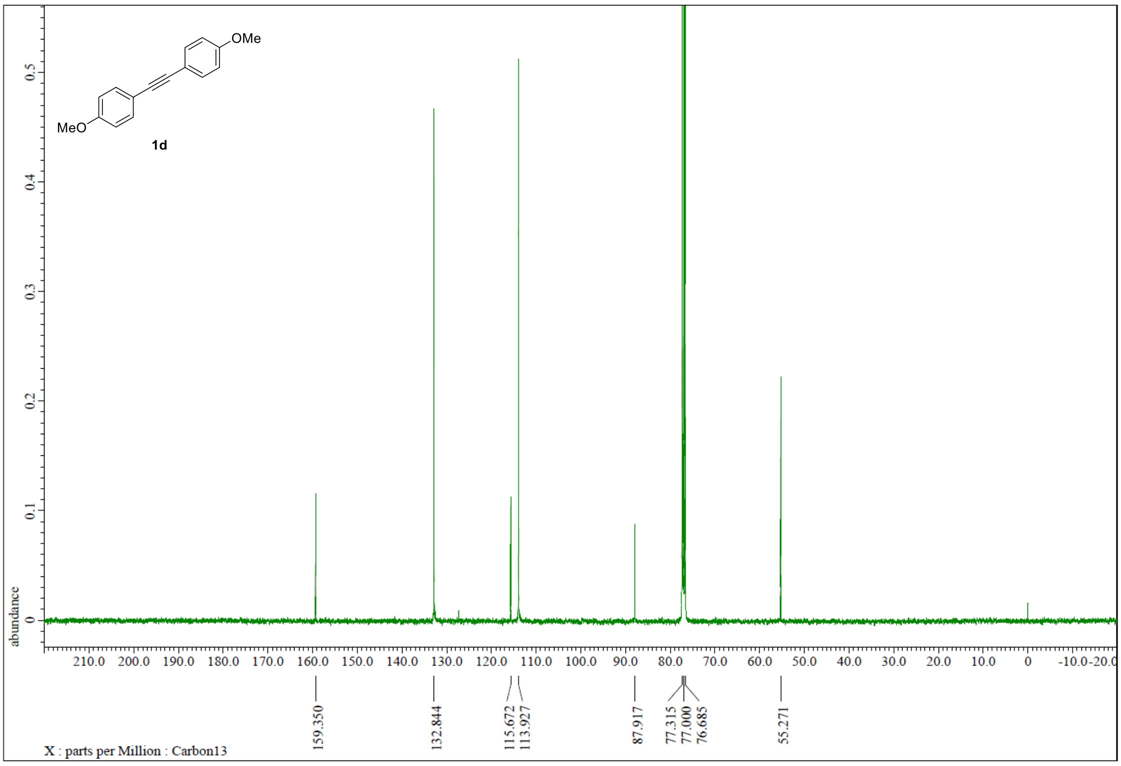

^{1}H NMR (400 MHz, $CDCl_3$) of **1e**

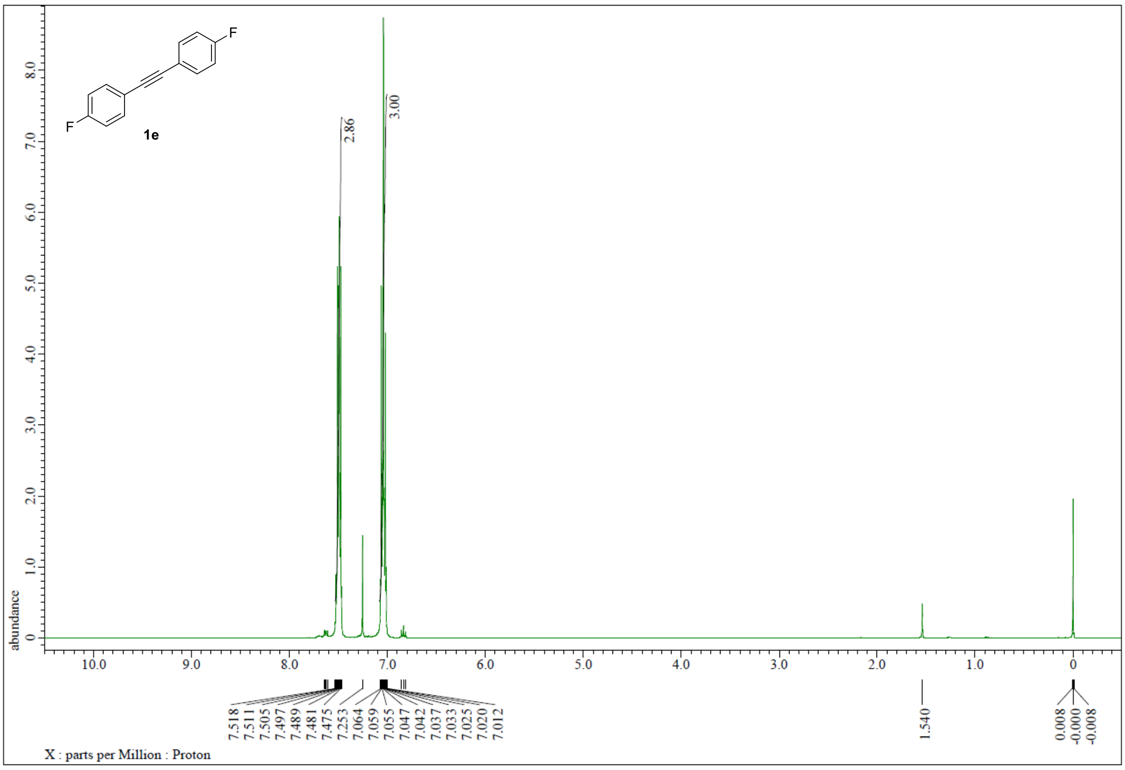

^{13}C NMR (100 MHz, $CDCl_3$) of **1e**. A small amount of impurity was confirmed.

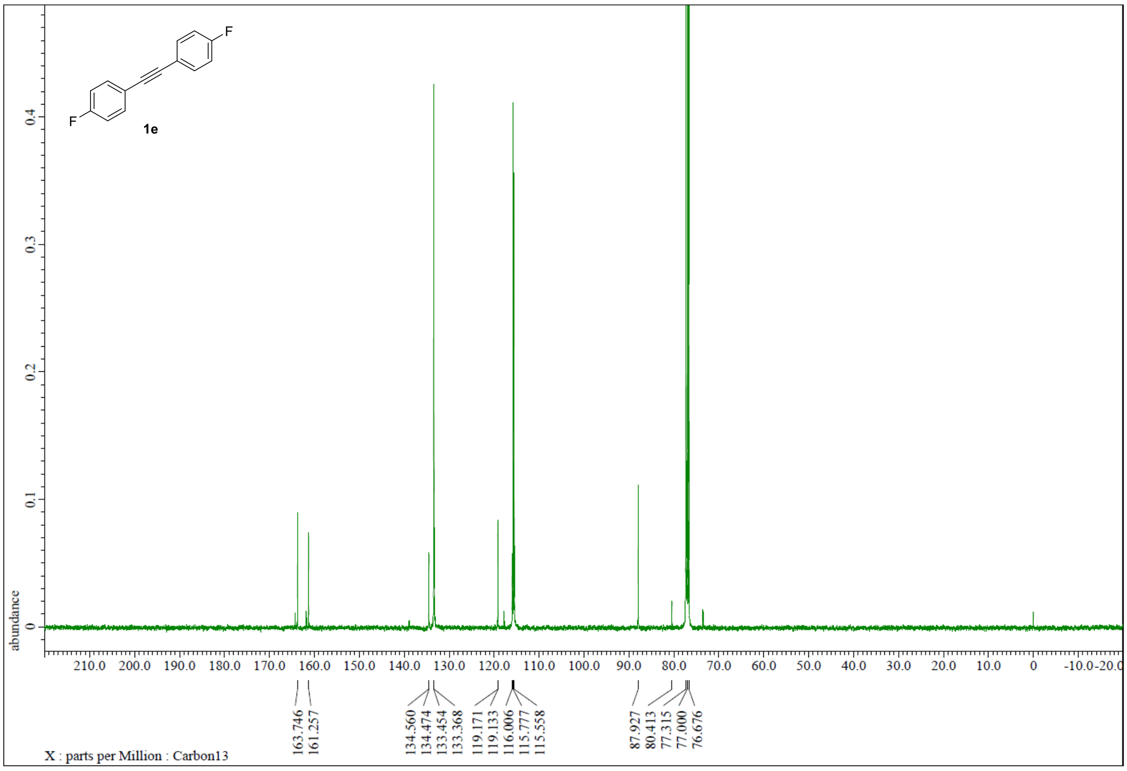

^{1}H NMR (400 MHz, $CDCl_3$) of **1g**

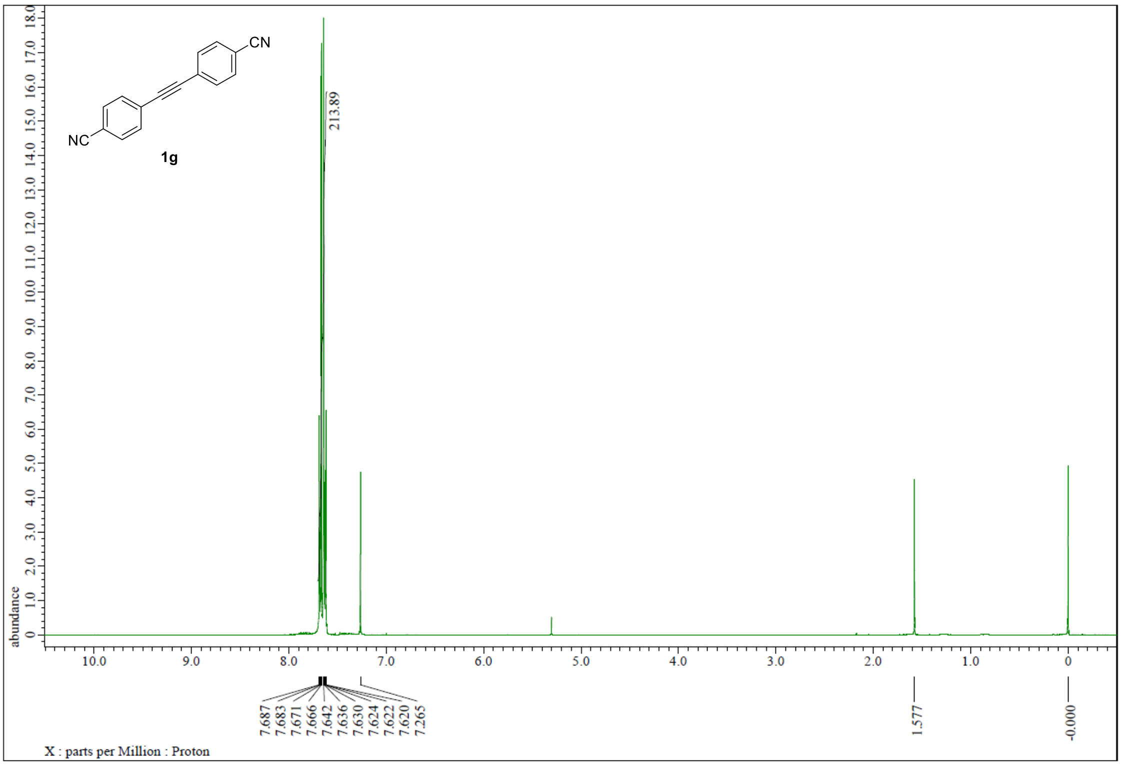

^{13}C NMR (100 MHz, $CDCl_3$) of **1g**

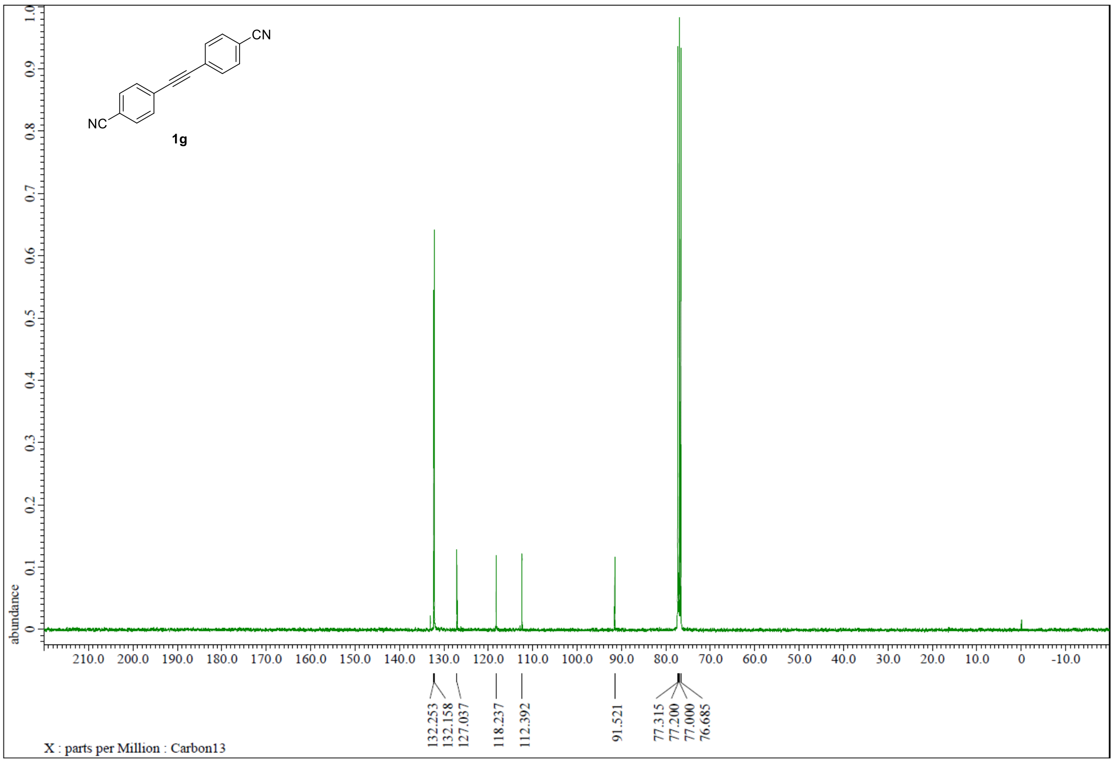

^{1}H NMR (400 MHz, $CDCl_3$) of **1h**

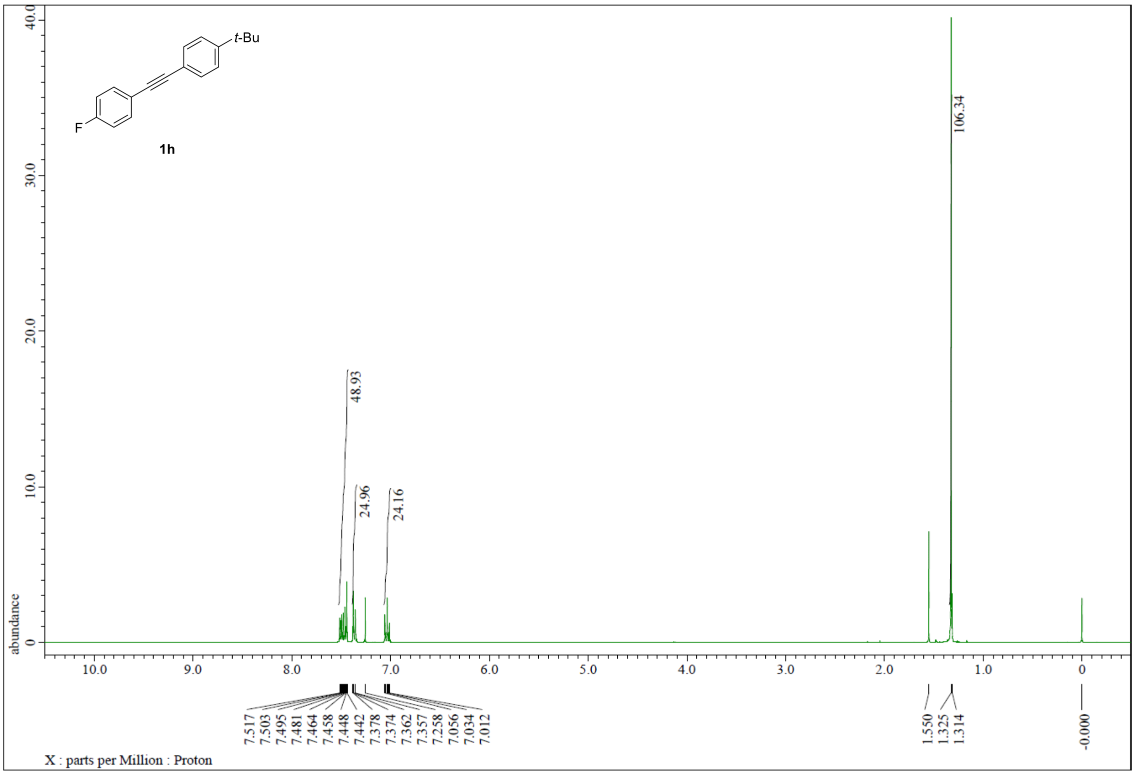

^{13}C NMR (100 MHz, $CDCl_3$) of **1h**

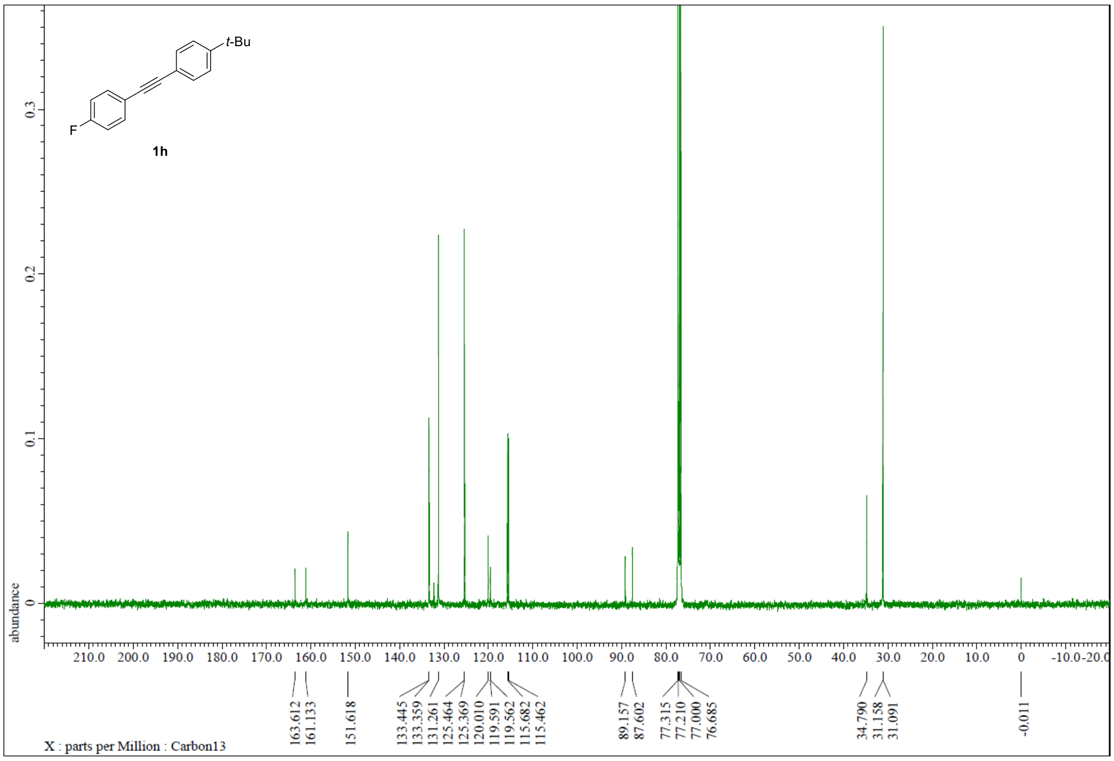

^{1}H NMR (400 MHz, $CDCl_3$) of ***E*-2a** (Table 1, entry 1)

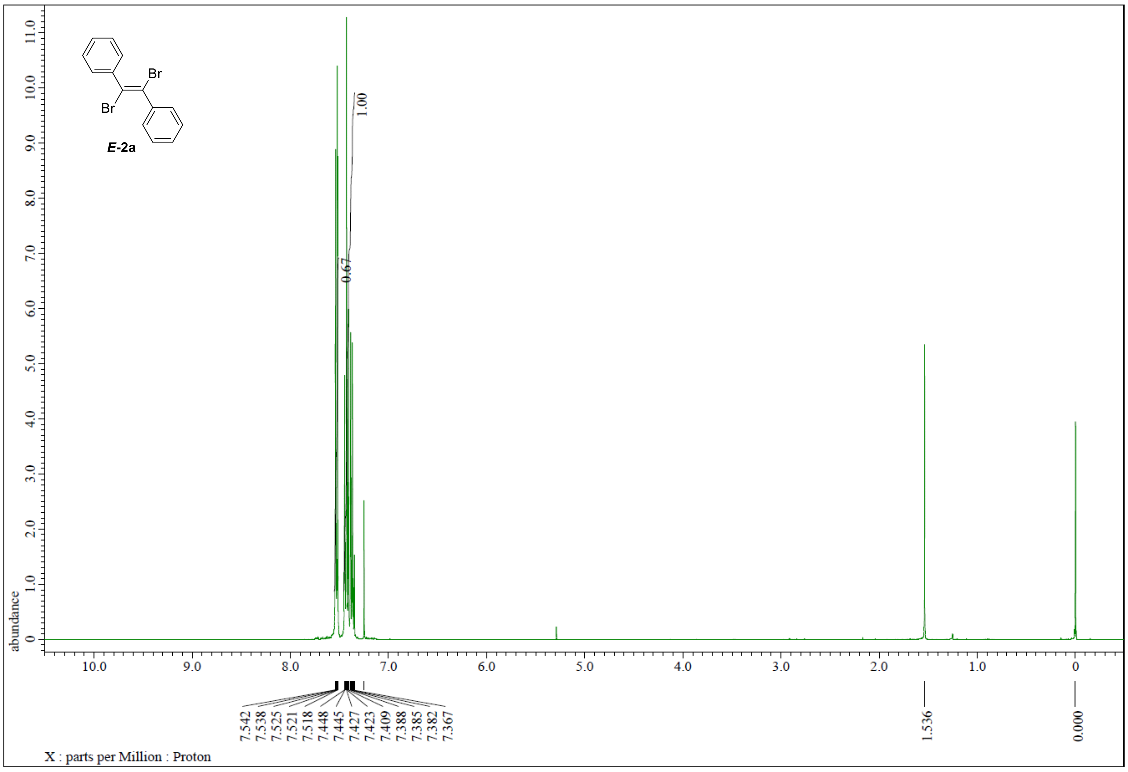

^{13}C NMR (100 MHz, $CDCl_3$) of ***E*-2a** (Table 1, entry 1)

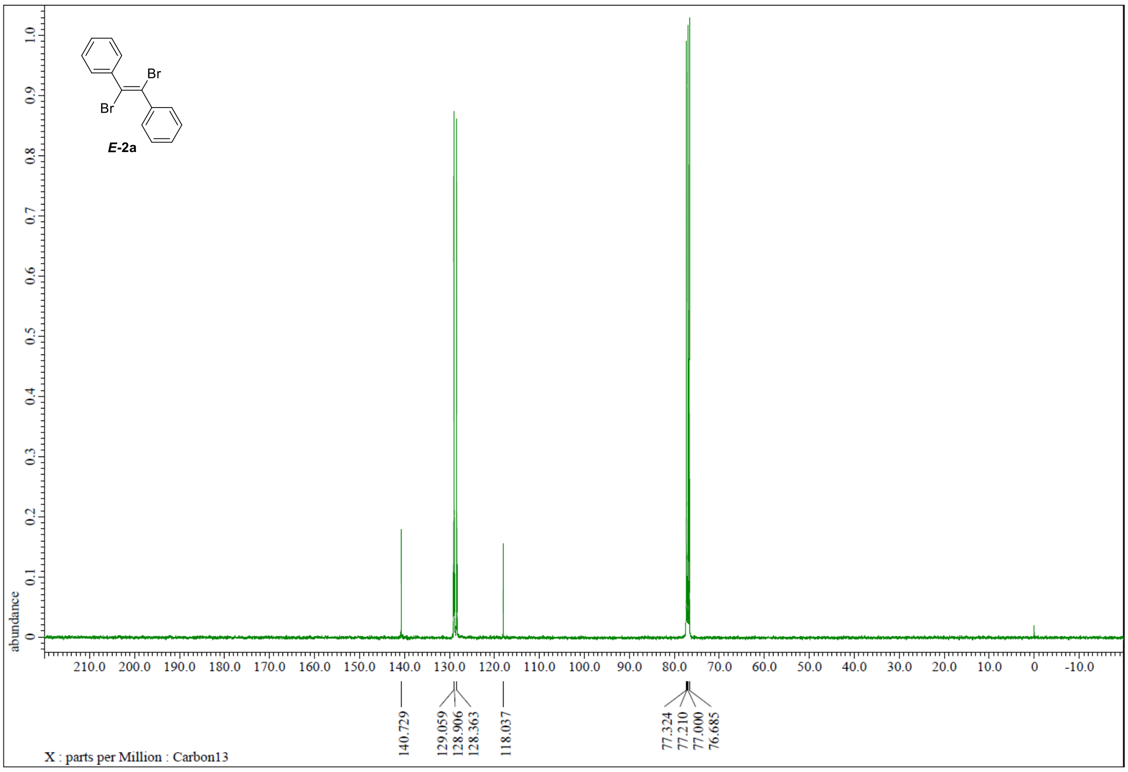

^{1}H NMR (300 MHz, $CDCl_3$) of ***Z*-2a** (Table 1, entry 1)

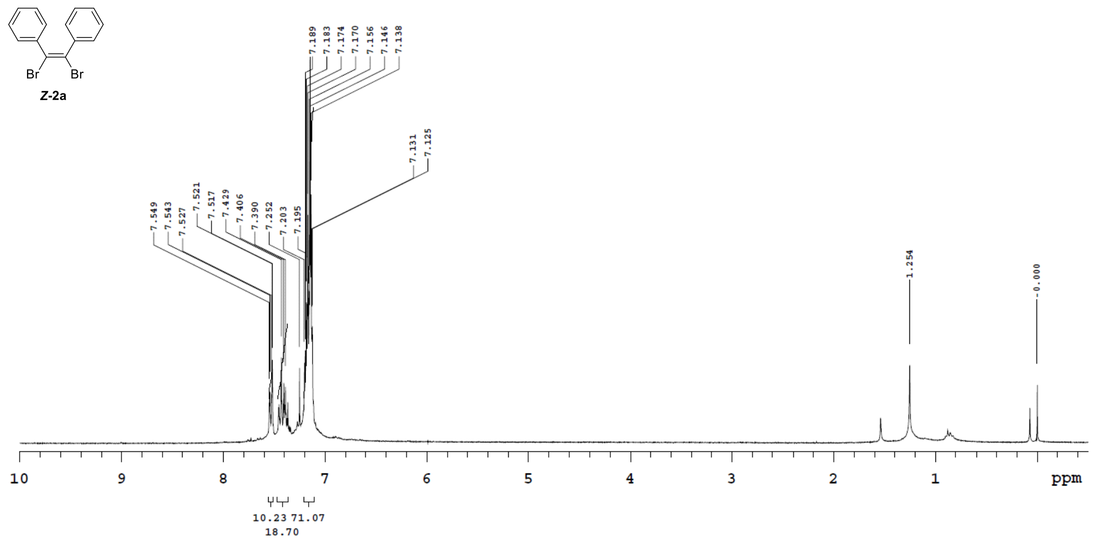

^{13}C NMR (75 MHz, $CDCl_3$) of ***Z*-2a** (Table 1, entry 1)

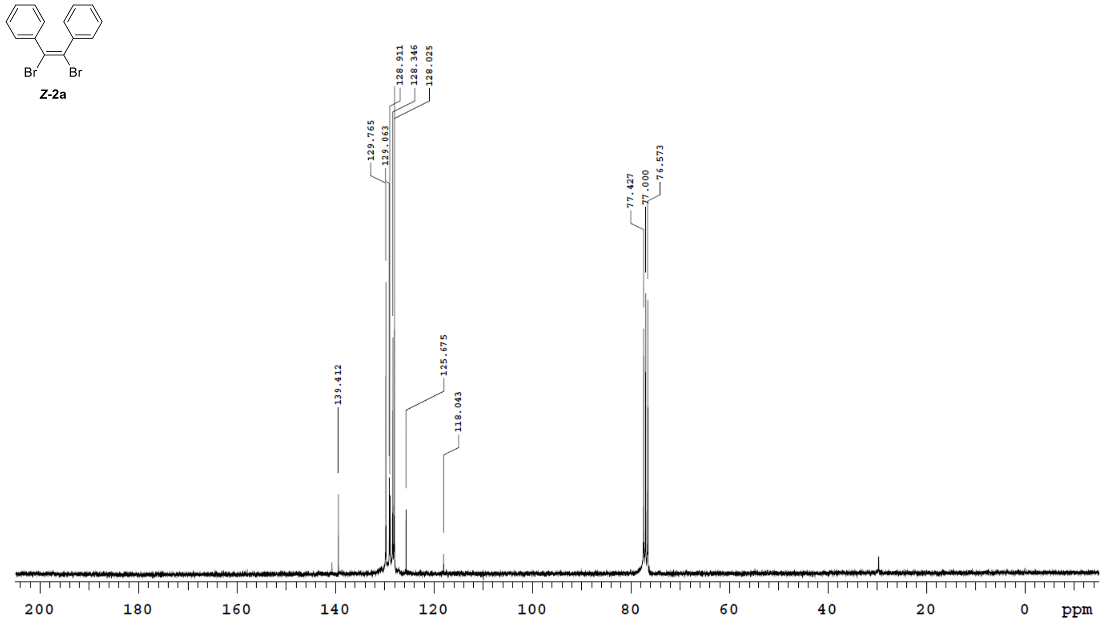

^{1}H NMR (400 MHz, $CDCl_3$) of ***E*-2b** (Table 2, entry 1).

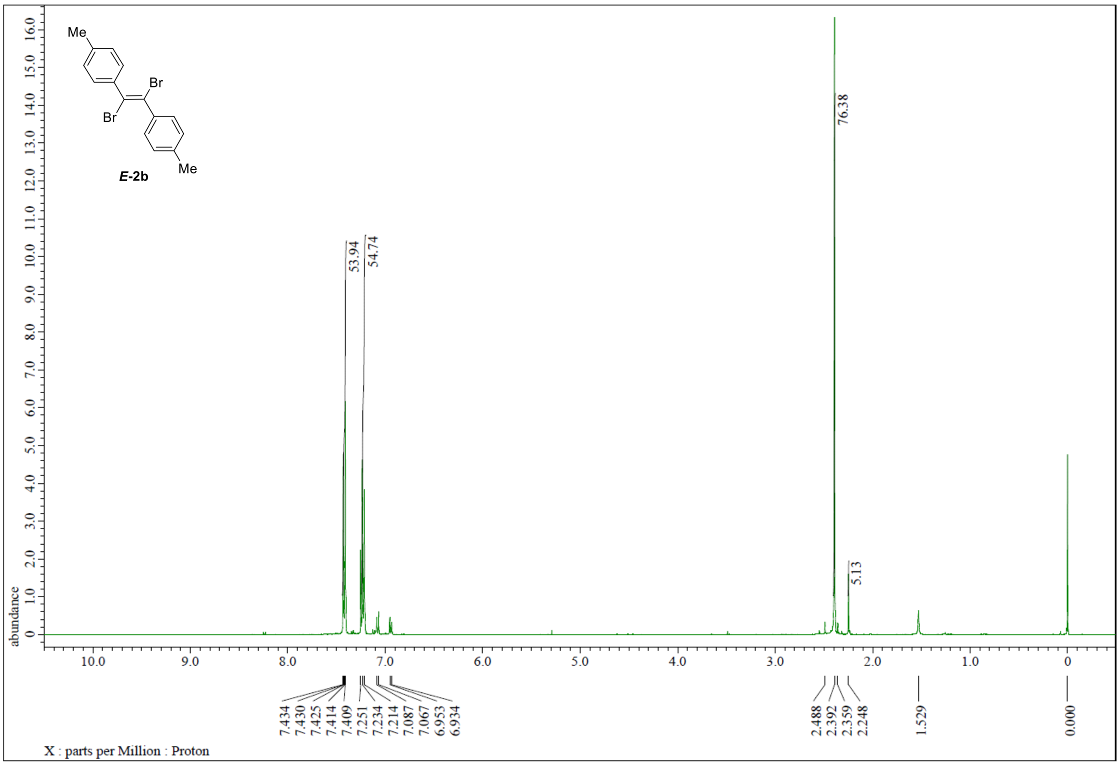

^{13}C NMR (100 MHz, $CDCl_3$) of ***E*-2b** (Table 2, entry 1)

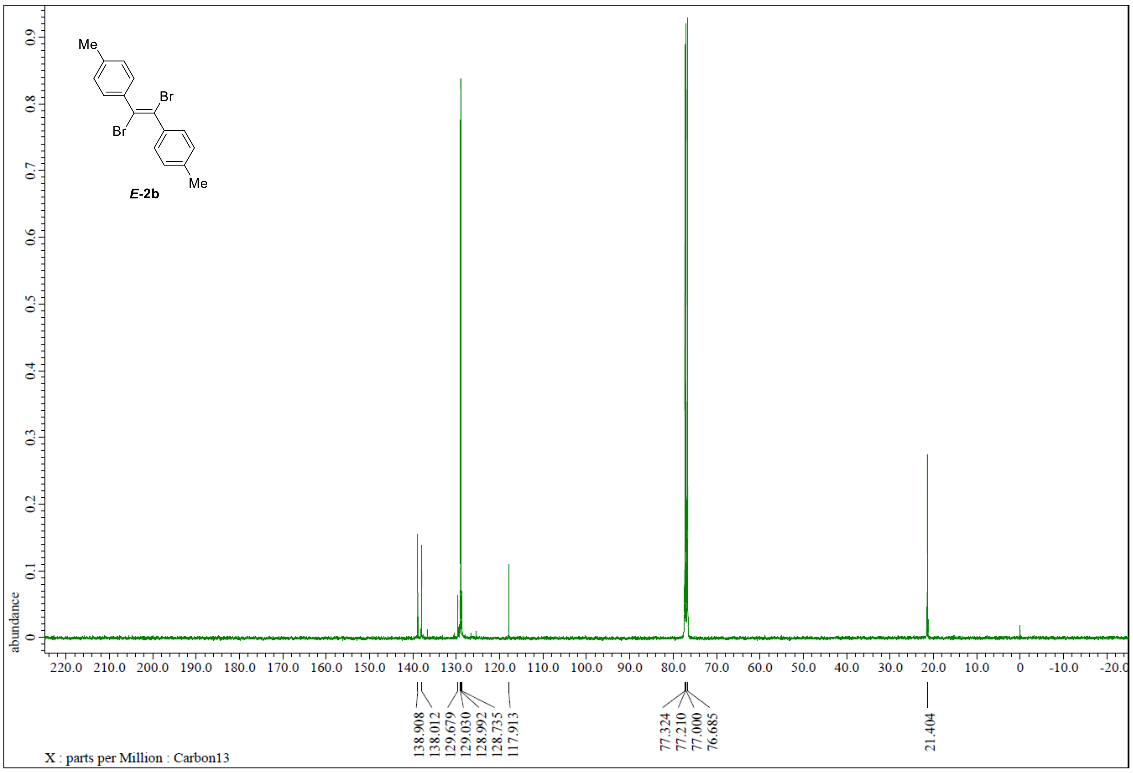

^{1}H NMR (400 MHz, CDCl3) of ***Z*-2b** (Table 2, entry 1)

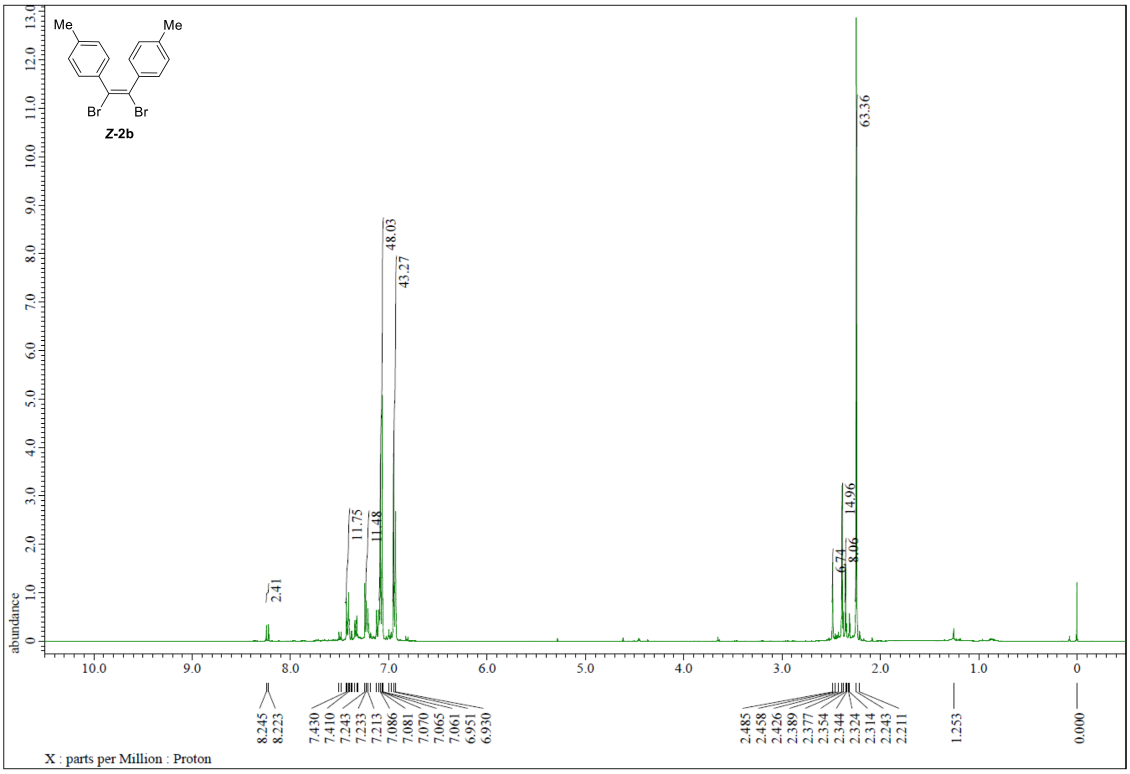

^{13}C NMR (100 MHz, CDCl3) of ***Z*-2b** (Table 2, entry 1)

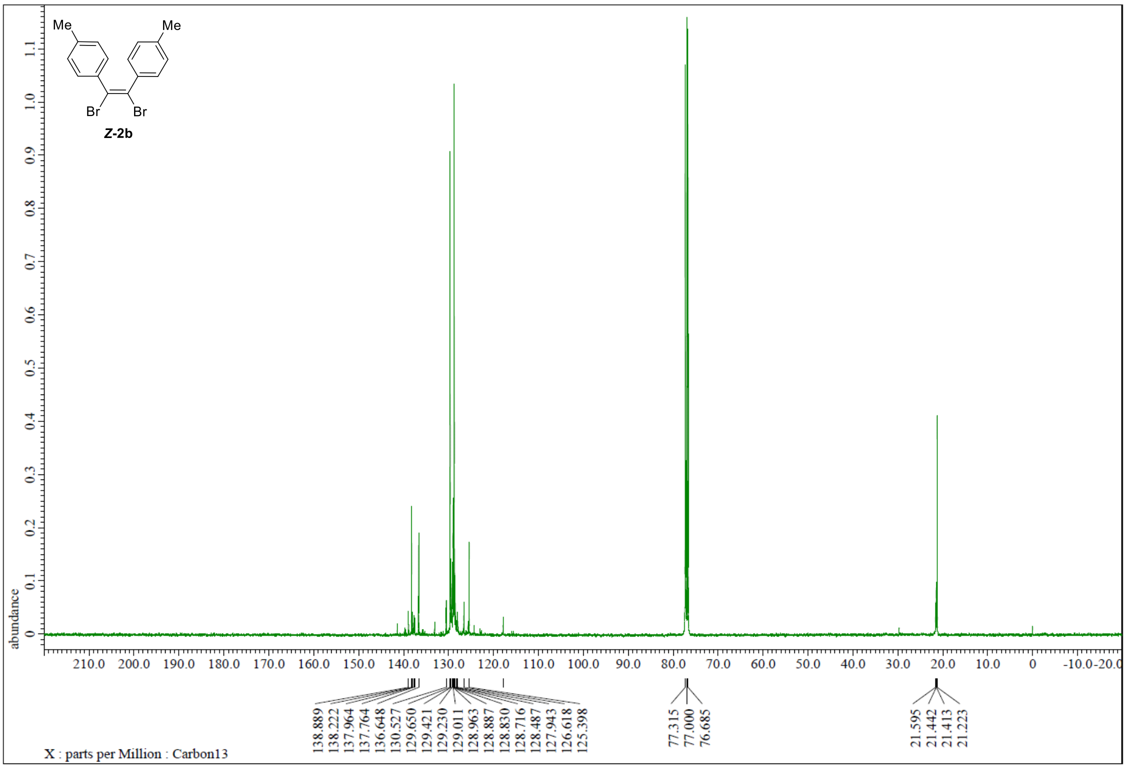

^{1}H NMR (400 MHz, CDCl3) of ***E*-2c** (Table 2, entry 2)

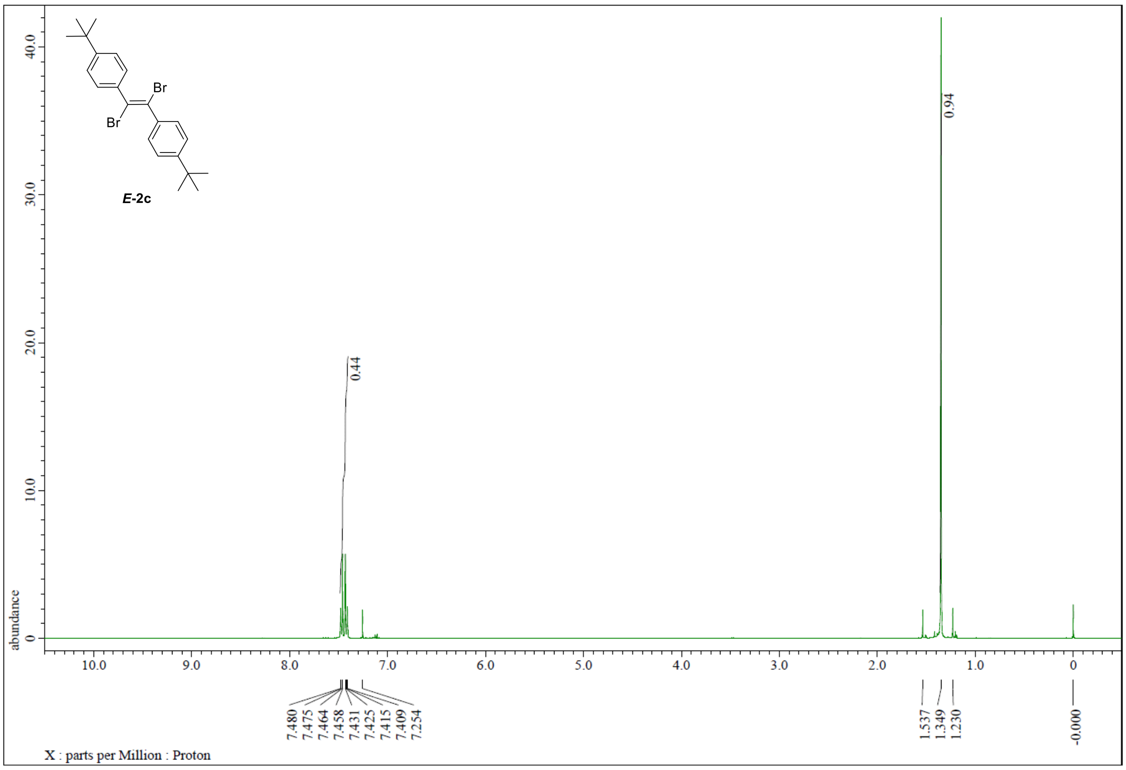

^{13}C NMR (100 MHz, CDCl3) of ***E*-2c** (Table 2, entry 2)

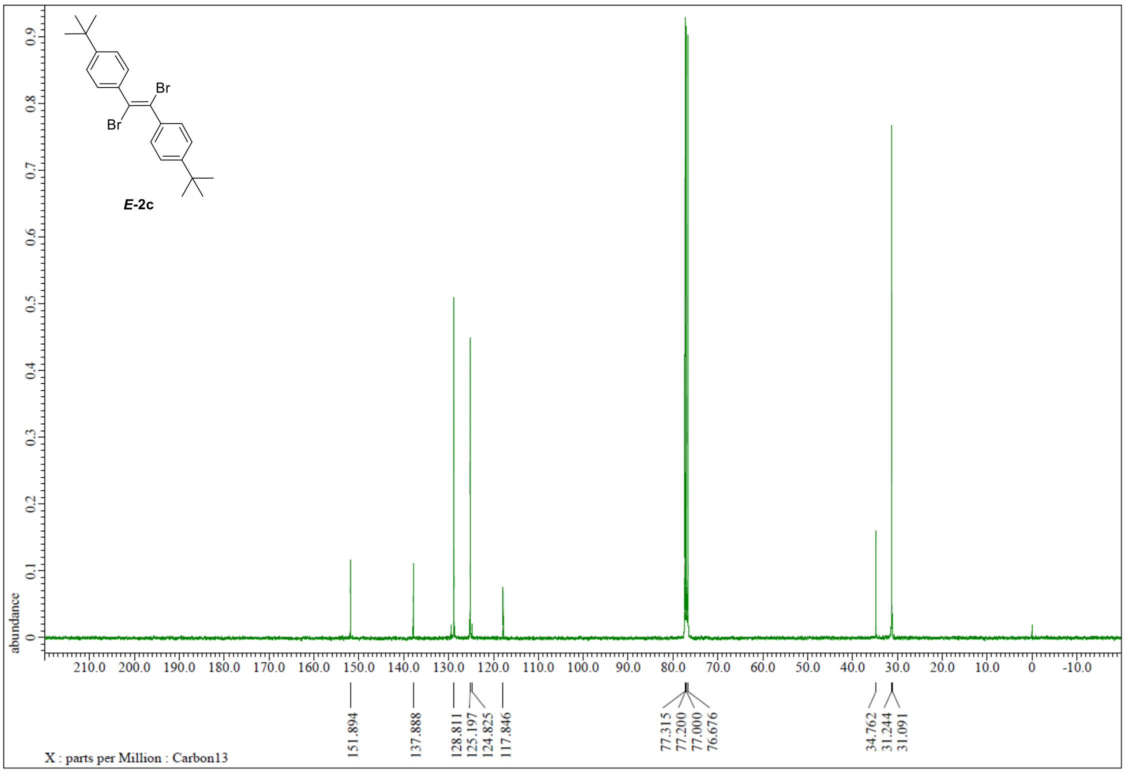

^{1}H NMR (400 MHz, $CDCl_3$) of ***Z*-2c** (Table 2, entry 2)

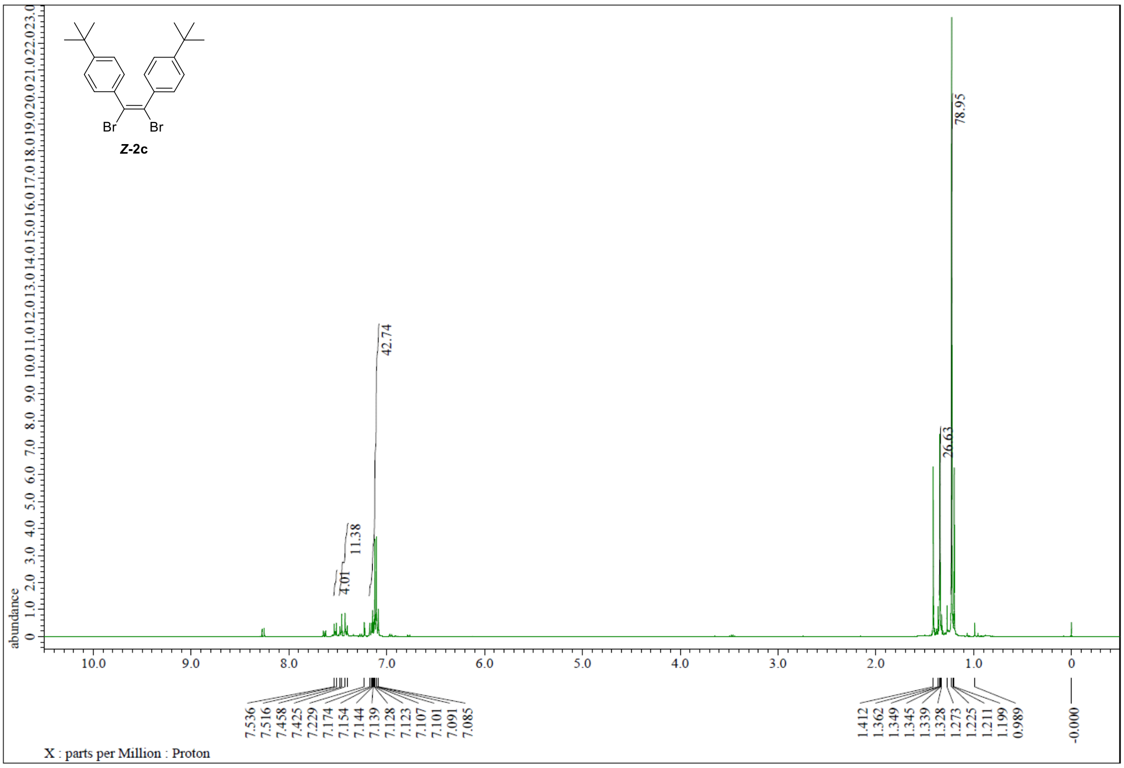

^{13}C NMR (100 MHz, $CDCl_3$) of ***Z*-2c** (Table 2, entry 2)

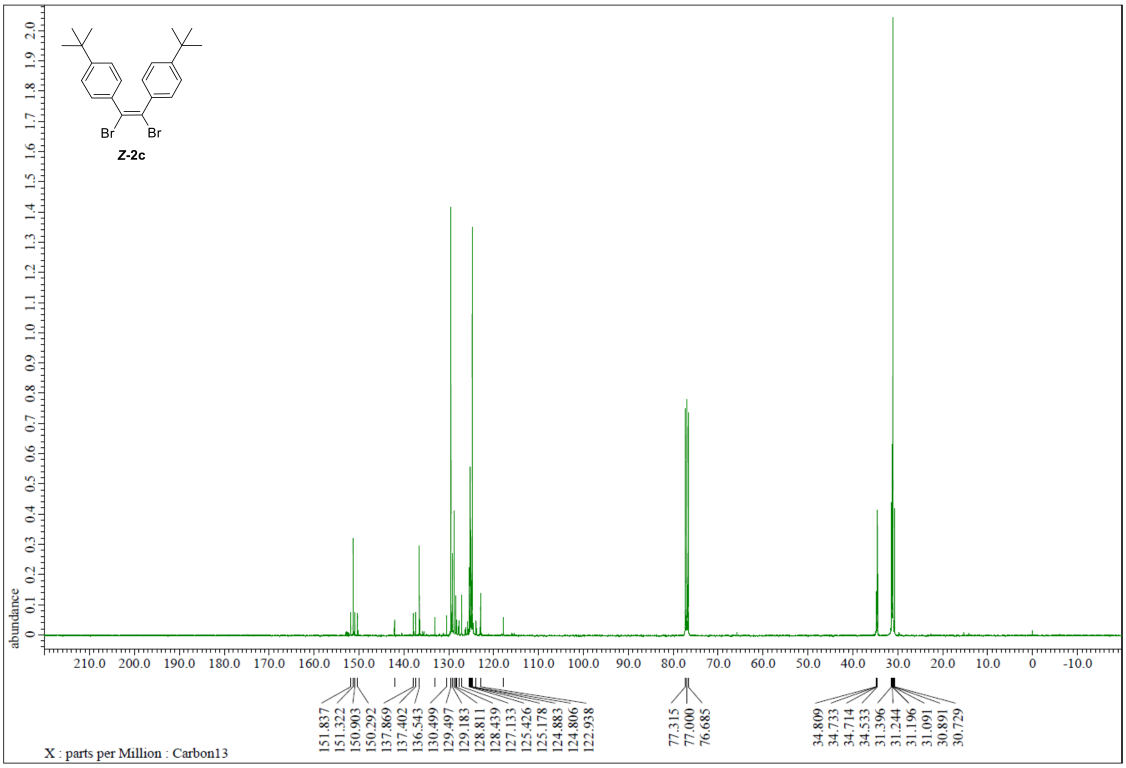

^{1}H NMR (300 MHz, $CDCl_3$) of ***E*-2d** (Table 2, entry 3)

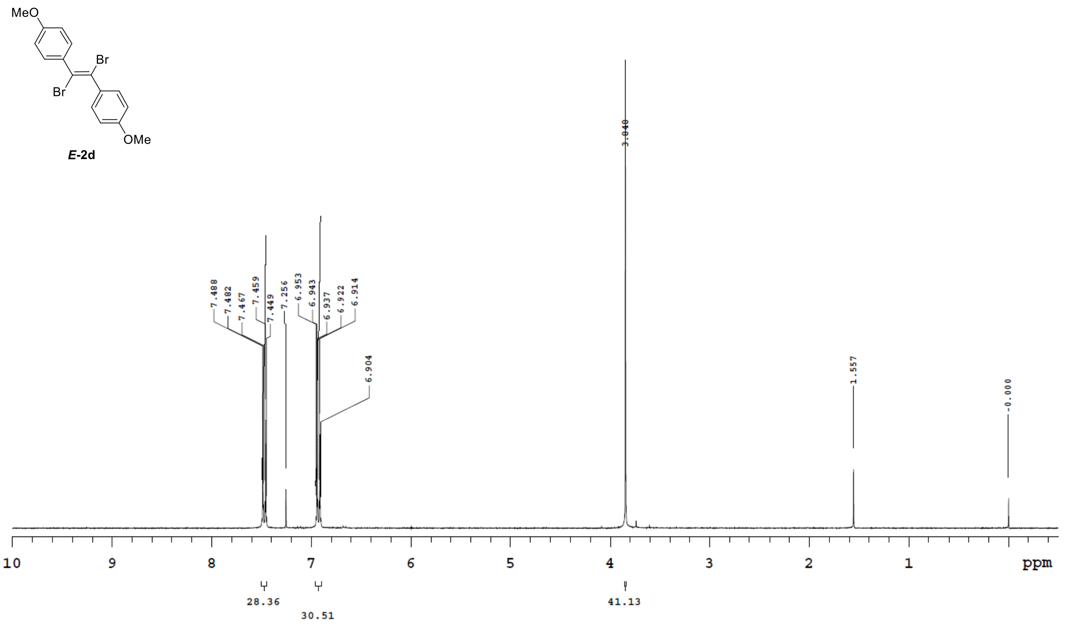

^{13}C NMR (75 MHz, $CDCl_3$) of ***E*-2d** (Table 2, entry 3)

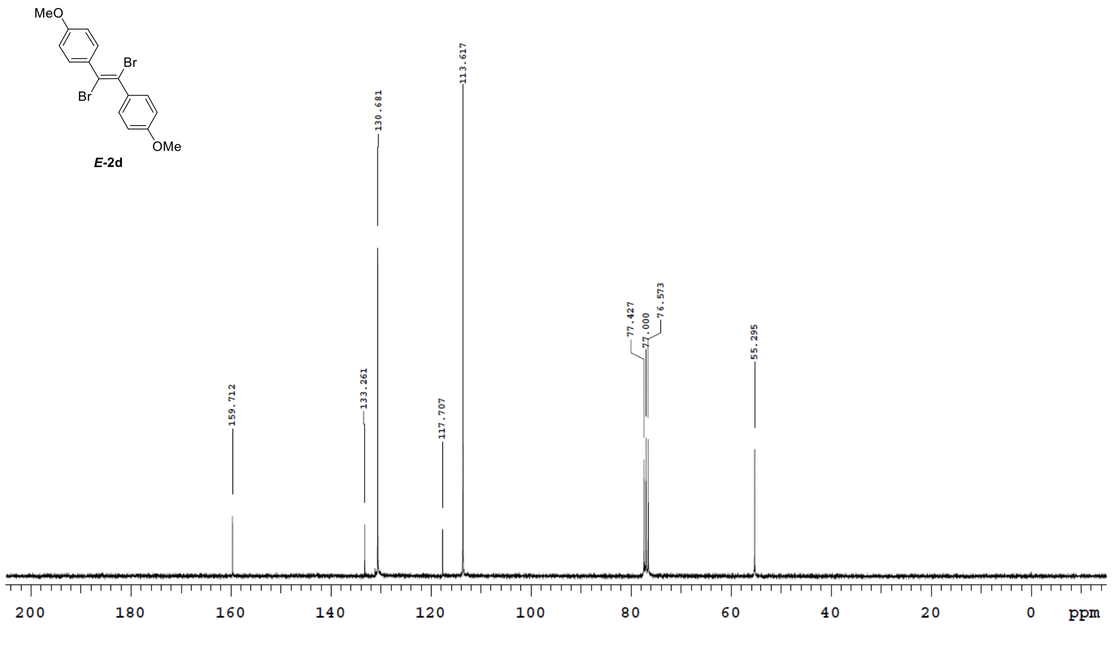

^{1}H NMR (400 MHz, $CDCl_3$) of ***Z*-2d** (Table 2, entry 3)

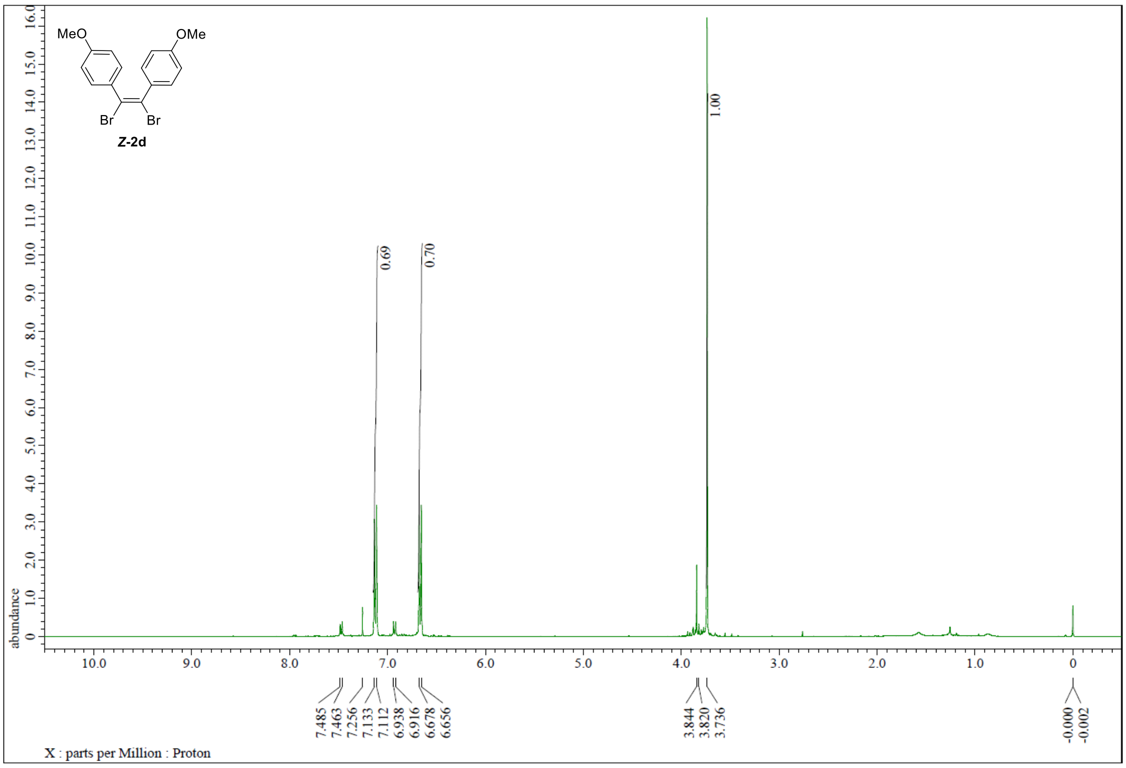

^{13}C NMR (100 MHz, $CDCl_3$) of ***Z*-2d** (Table 2, entry 3)

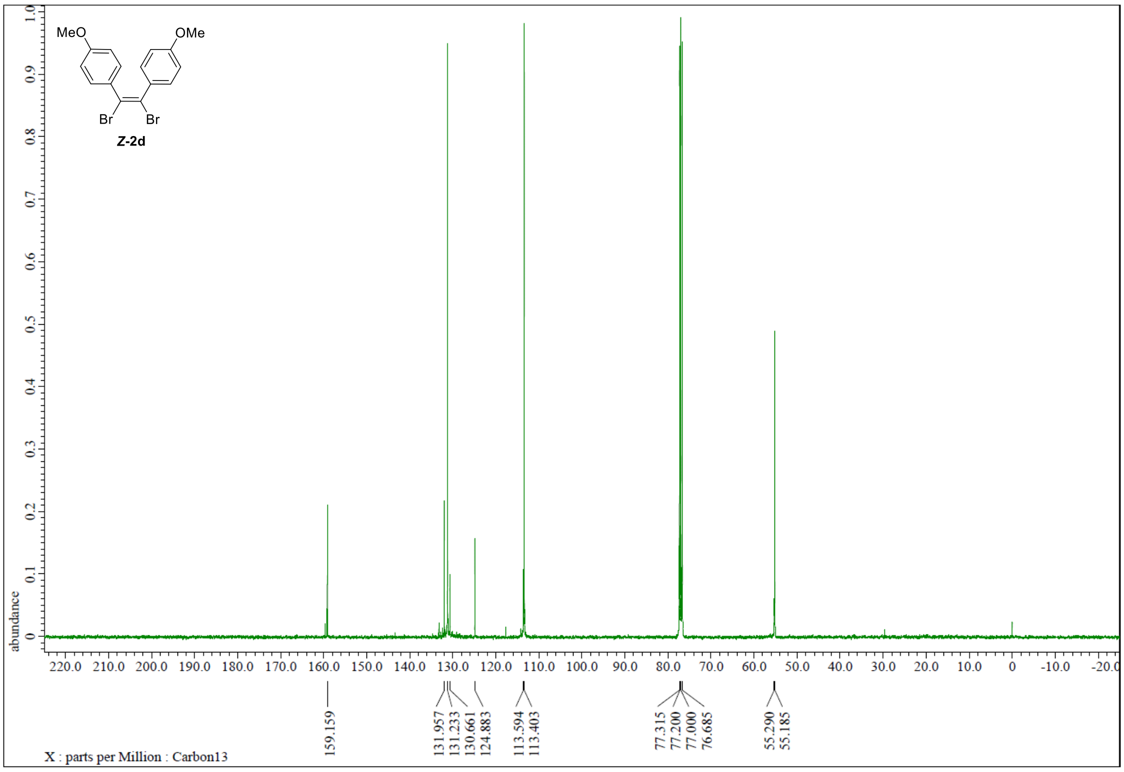

^{1}H NMR (400 MHz, $CDCl_3$) of ***E*-2e** (Table 2, entry 4)

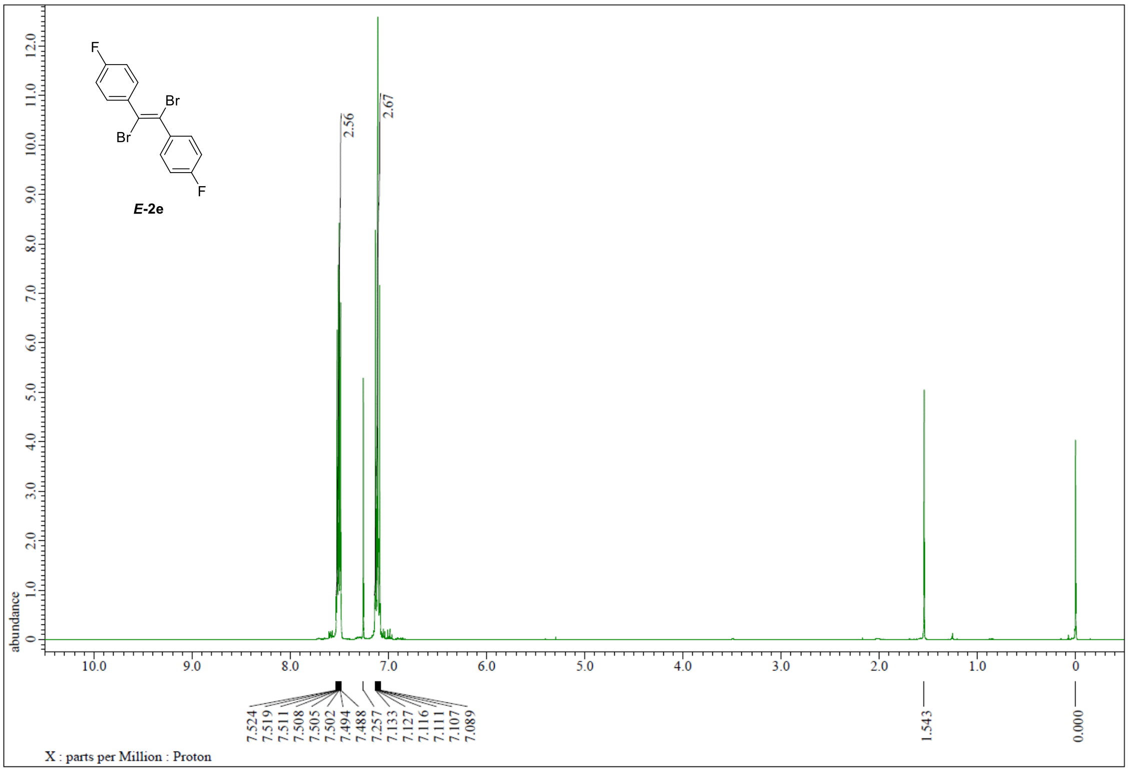

^{13}C NMR (100 MHz, $CDCl_3$) of ***E*-2e** (Table 2, entry 4)

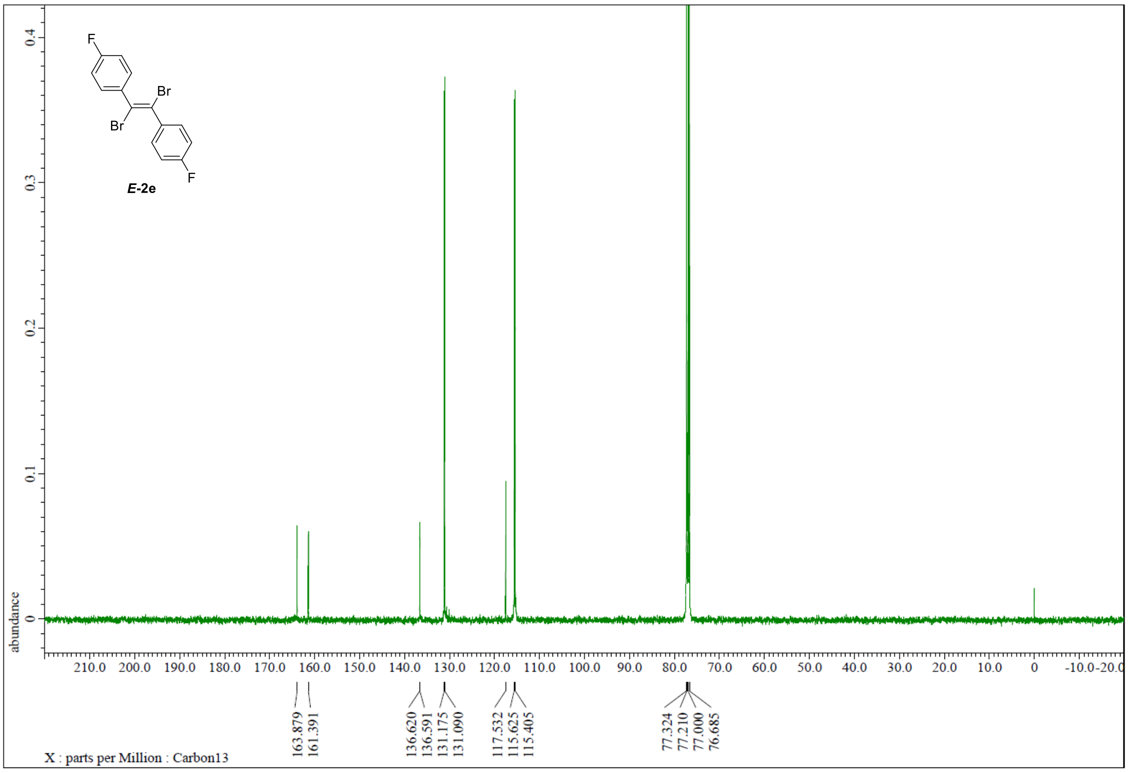

^{1}H NMR (400 MHz, CDCl$_3$) of ***Z*-2e** (Table 2, entry 4)

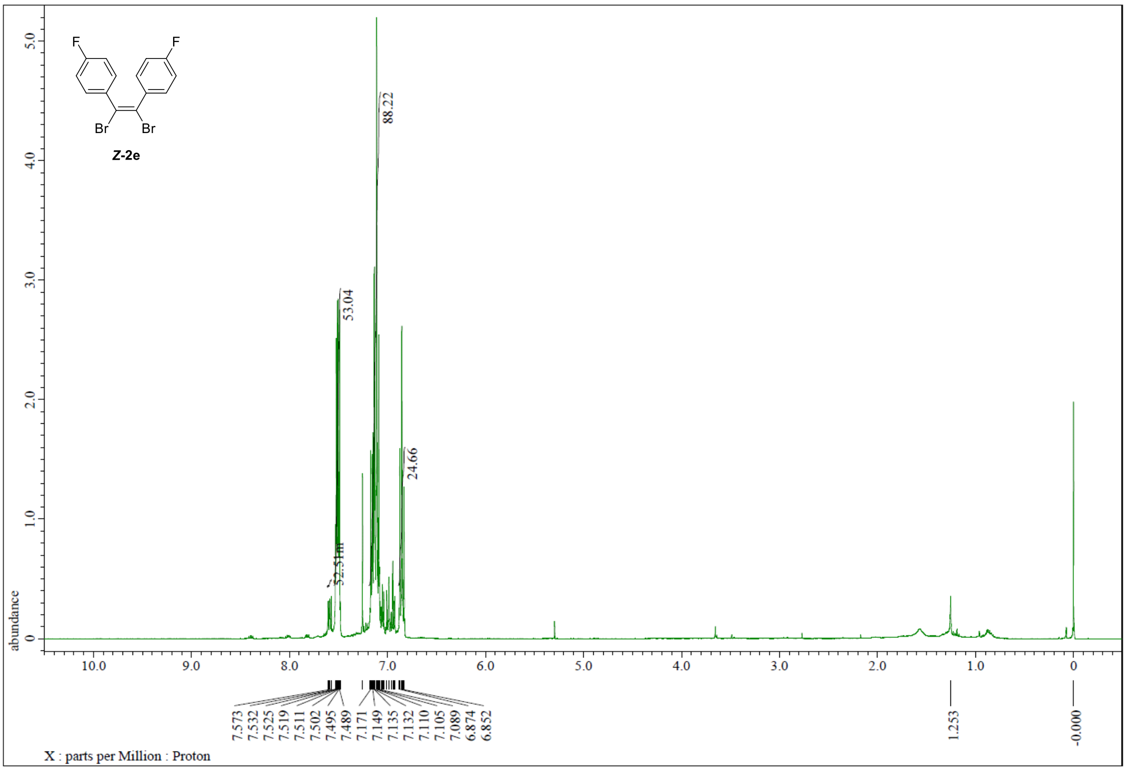

^{13}C NMR (100 MHz, CDCl$_3$) of ***Z*-2e** (Table 2, entry 4)

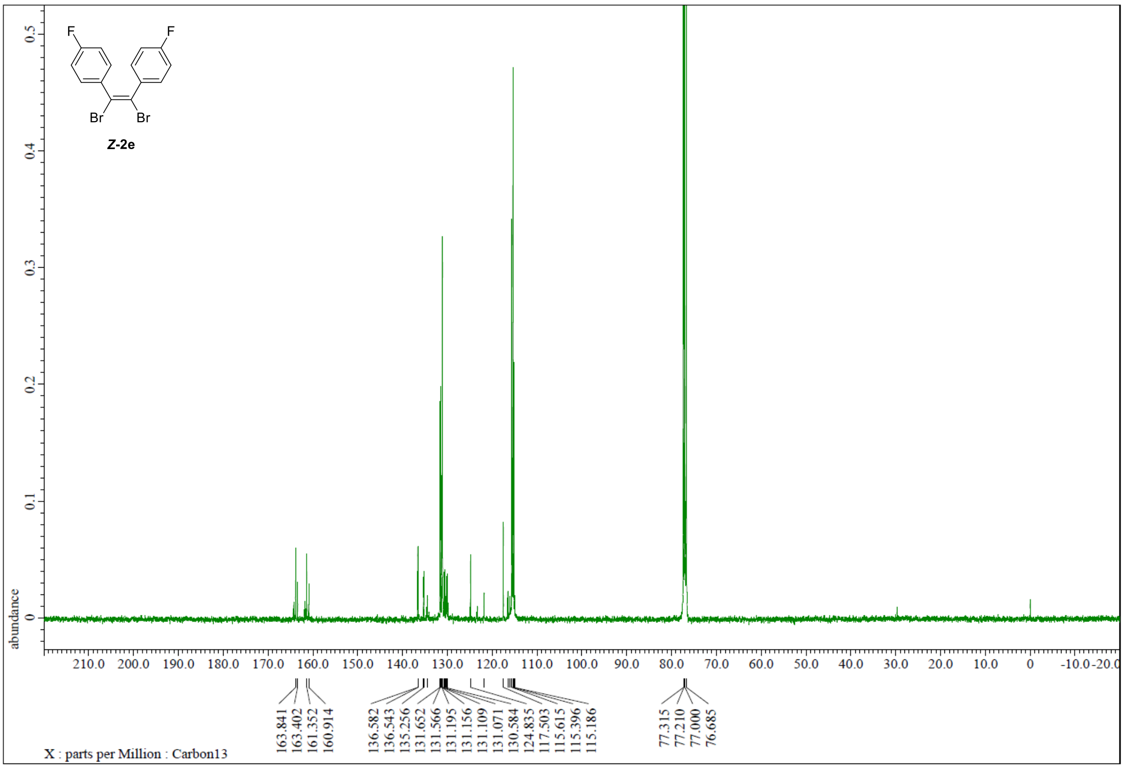

^{1}H NMR (400 MHz, CDCl3) of ***E*-2f** (Table 2, entry 5)

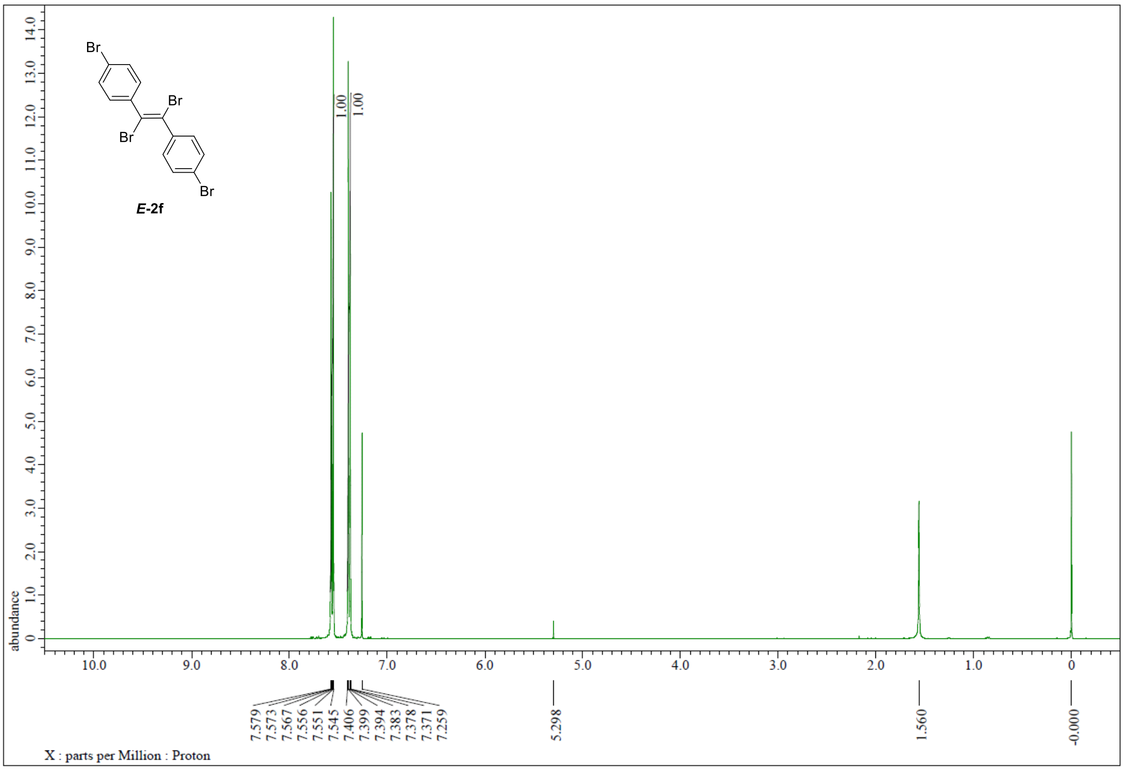

^{13}C NMR (100 MHz, CDCl3) of ***E*-2f** (Table 2, entry 5)

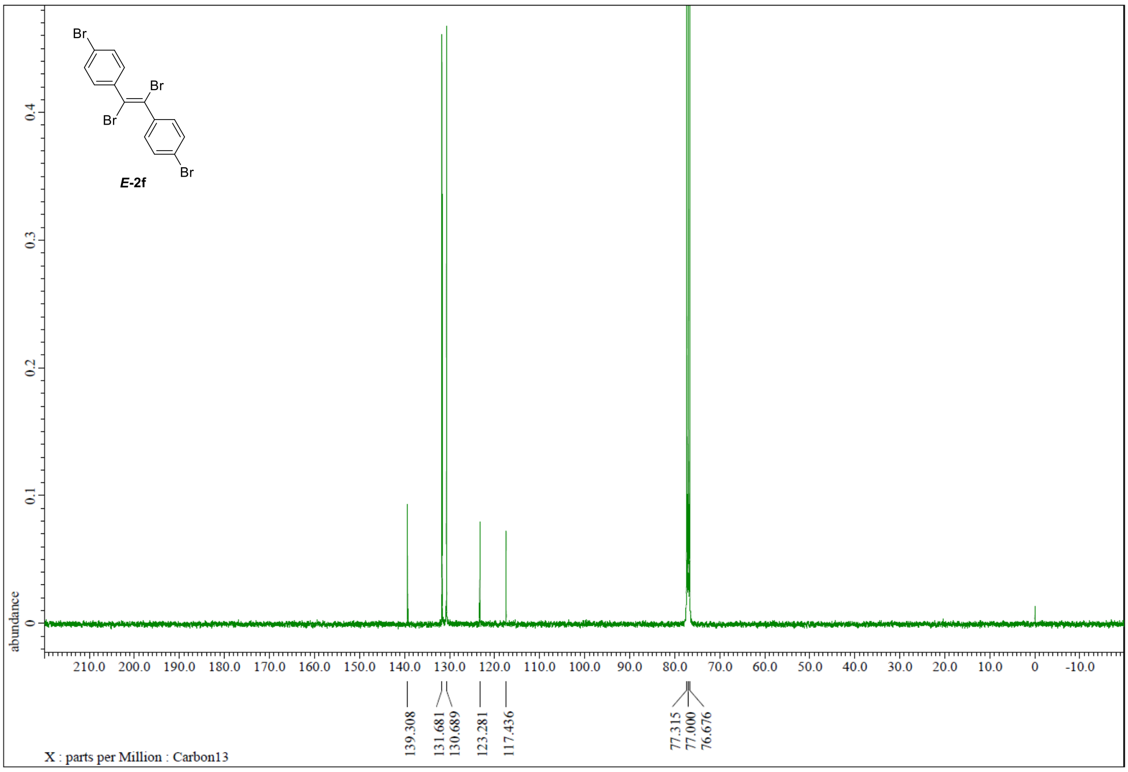

^1H NMR (400 MHz, $CDCl_3$) of ***Z*-2f** (Table 2, entry 5)

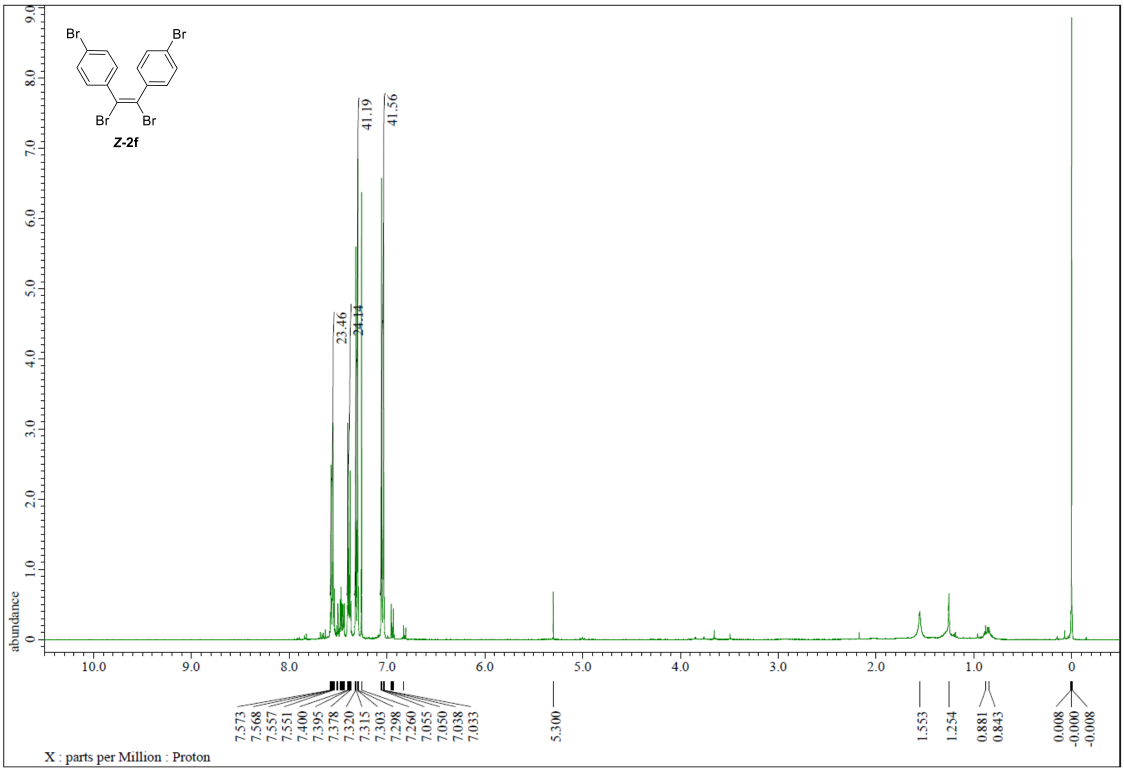

^{13}C NMR (100 MHz, $CDCl_3$) of ***Z*-2f** (Table 2, entry 5)

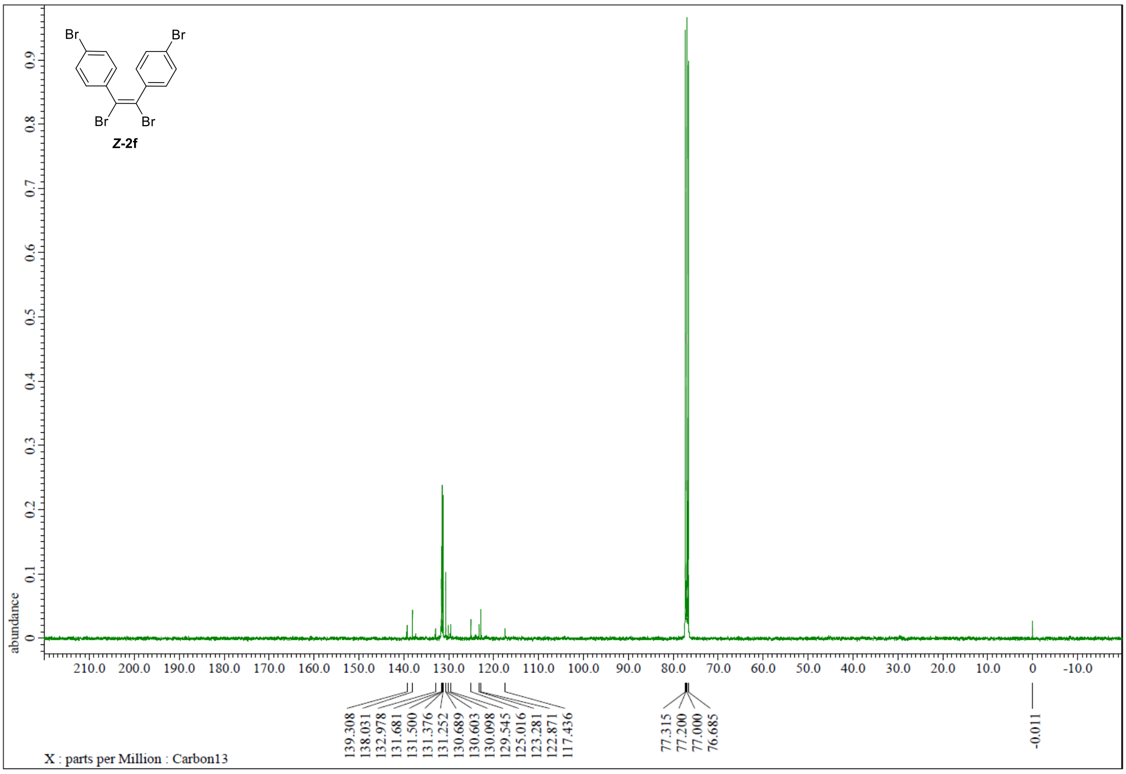

^{1}H NMR (400 MHz, $CDCl_3$) of ***E*-2g** (Table 2, entry 6)

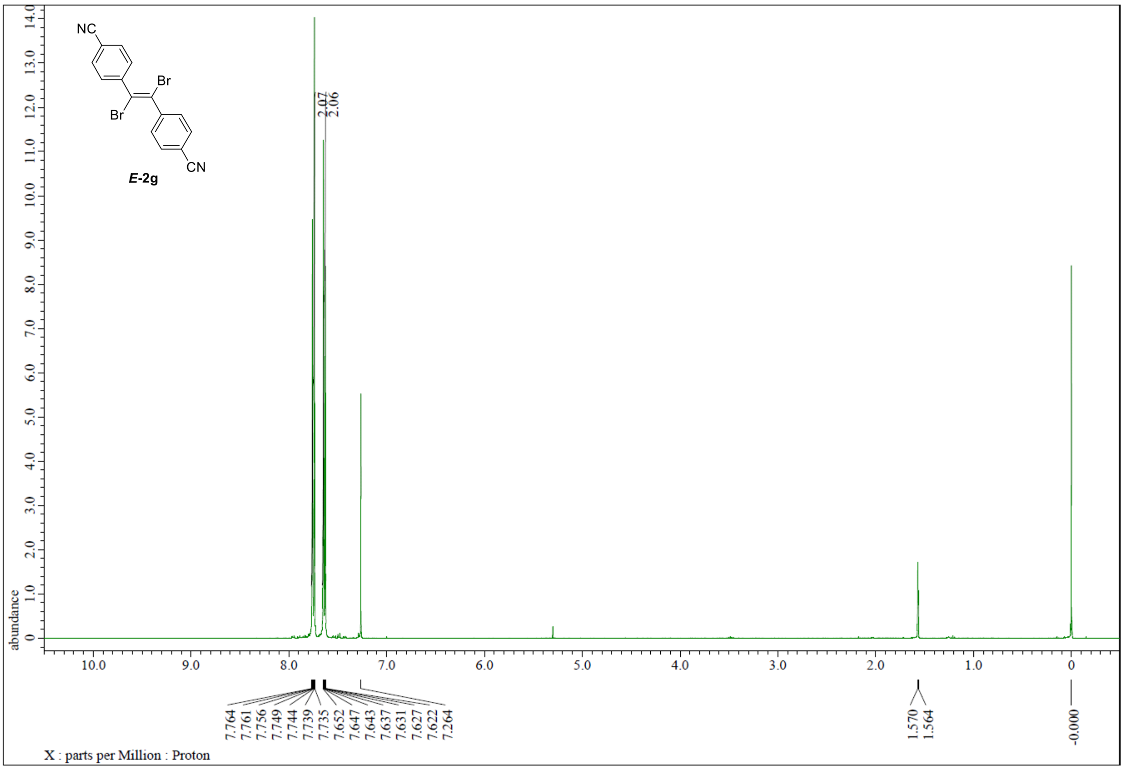

^{13}C NMR (100 MHz, $CDCl_3$) of ***E*-2g** (Table 2, entry 6)

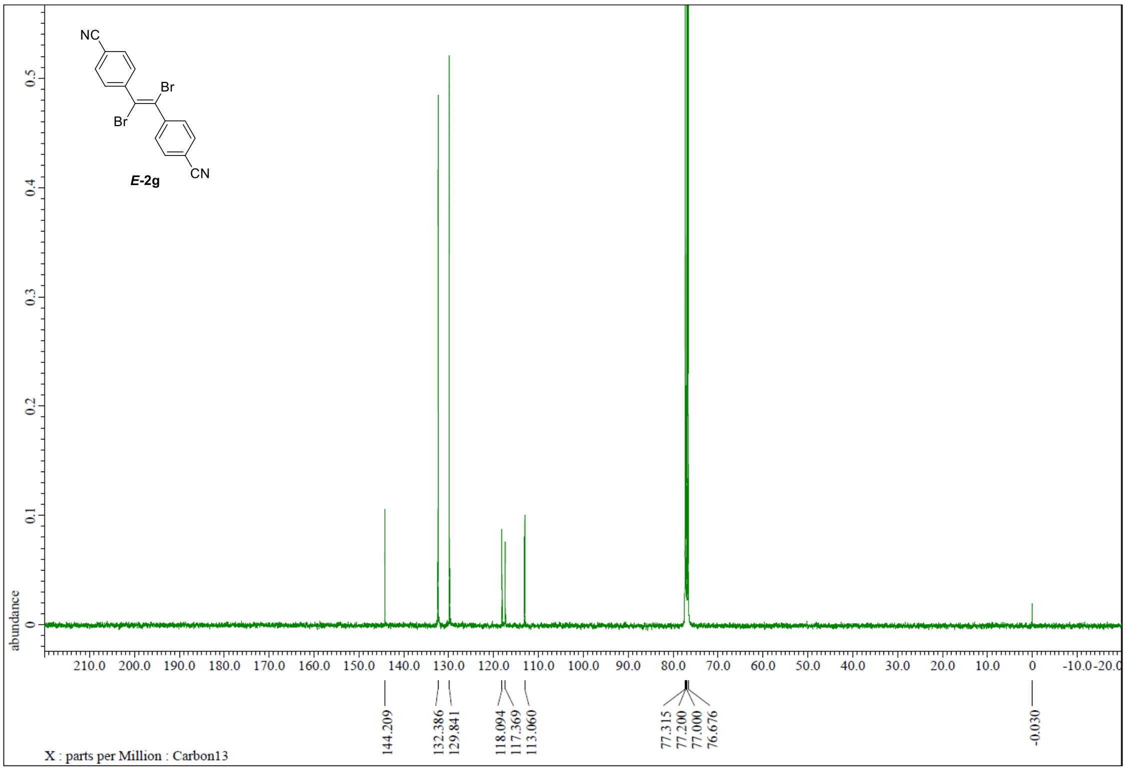

^{1}H NMR (400 MHz, $CDCl_3$) of ***Z*-2g** (Table 2, entry 6)

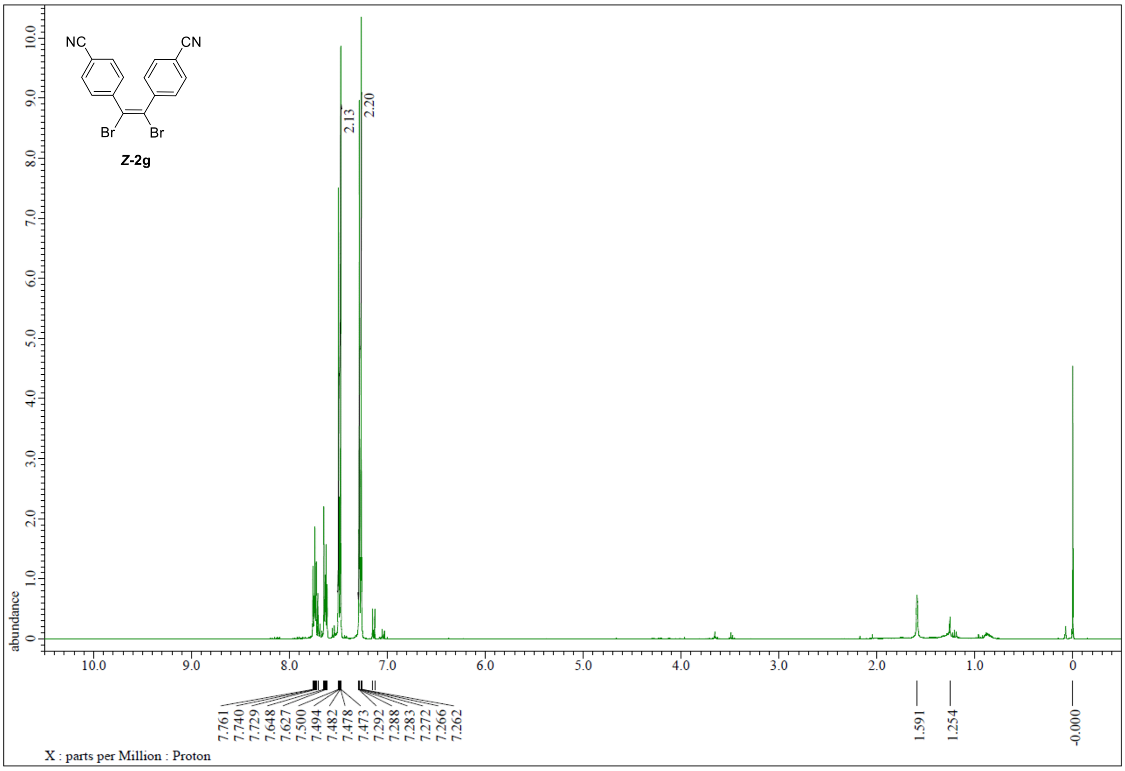

^{13}C NMR (100 MHz, $CDCl_3$) of ***Z*-2g** (Table 2, entry 6)

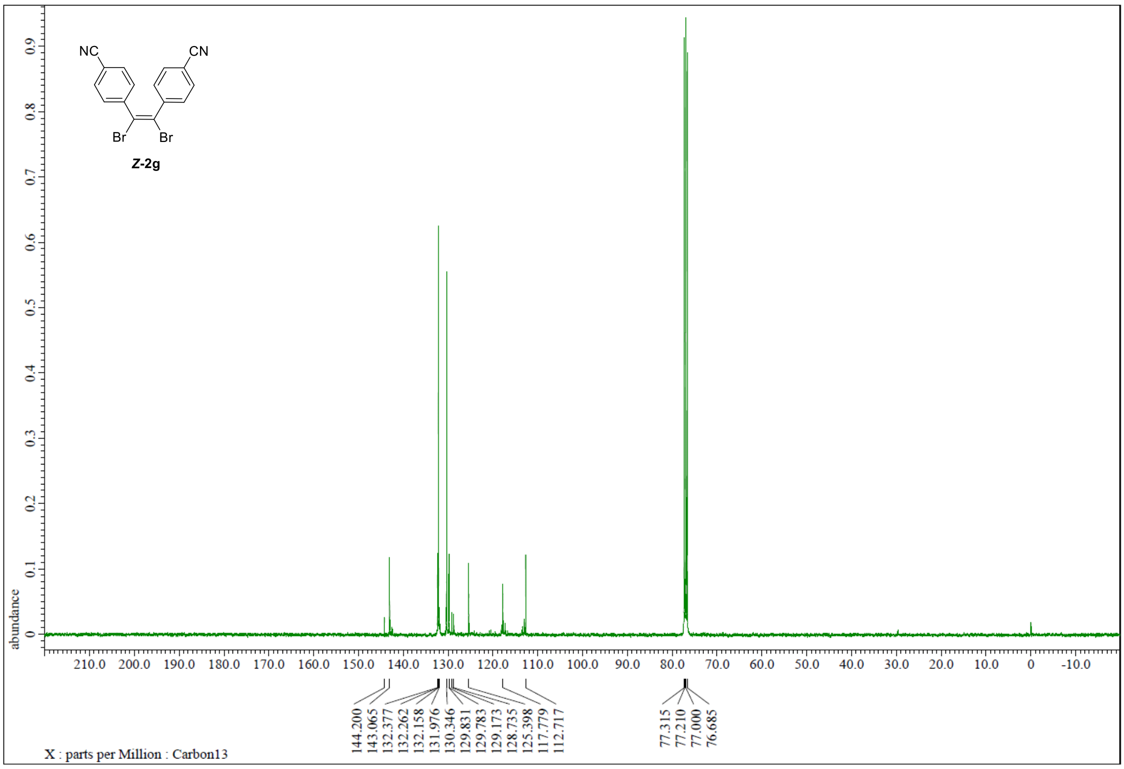

^{1}H NMR (400 MHz, $CDCl_3$) of ***E*-2h** (Table 2, entry 7)

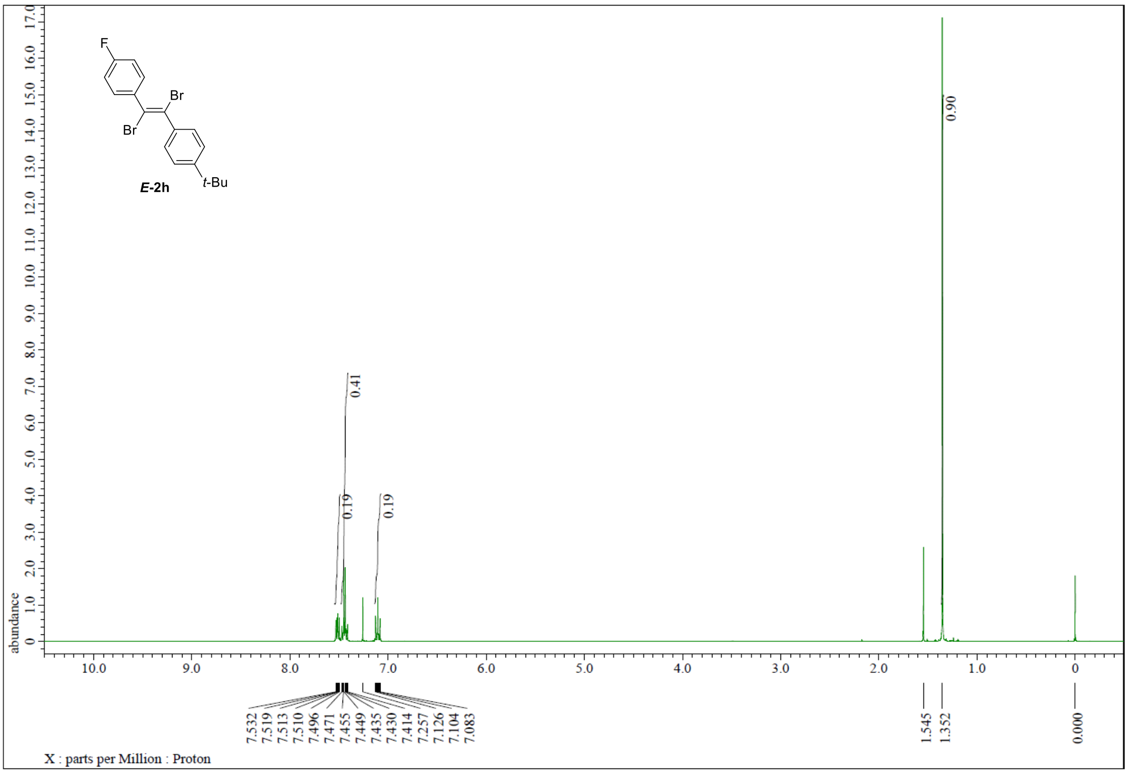

^{13}C NMR (100 MHz, $CDCl_3$) of ***E*-2h** (Table 2, entry 7)

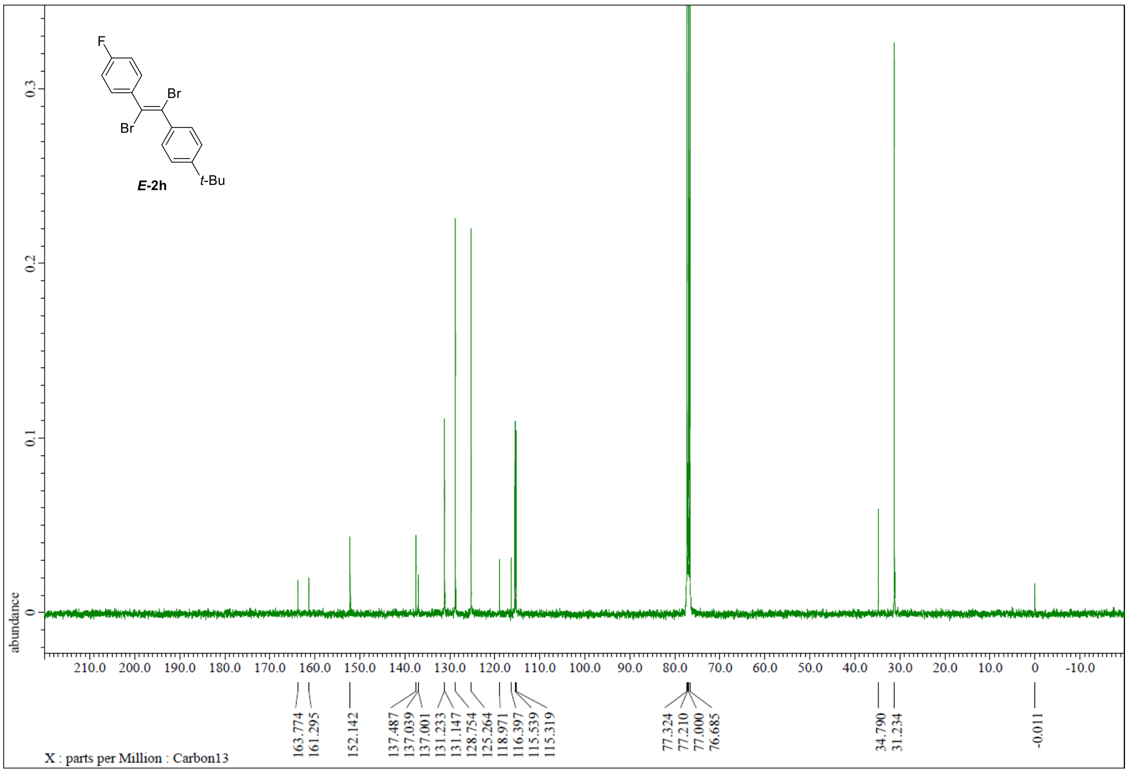

^{1}H NMR (400 MHz, CDCl$_3$) of ***Z*-2h** (Table 2, entry 7)

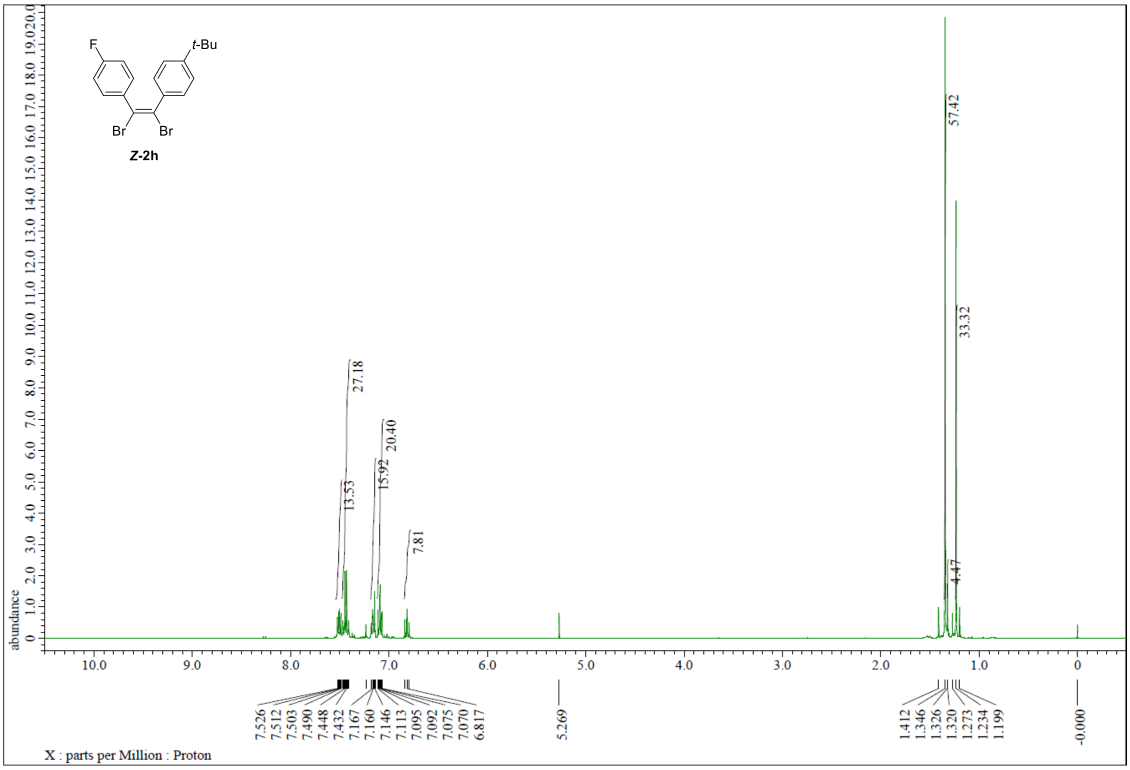

^{13}C NMR (100 MHz, CDCl$_3$) of ***Z*-2h** (Table 2, entry 7)

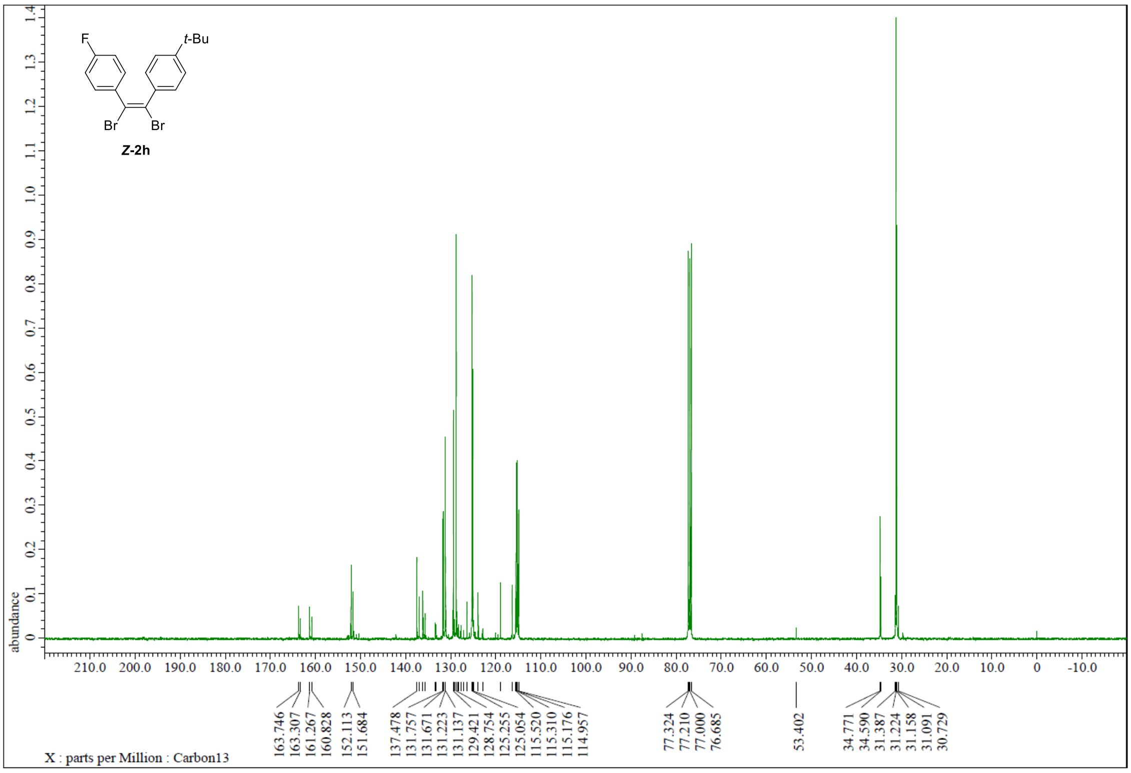

^{1}H NMR (400 MHz, CDCl3) of ***E*-2a** (Scheme 2 (a))

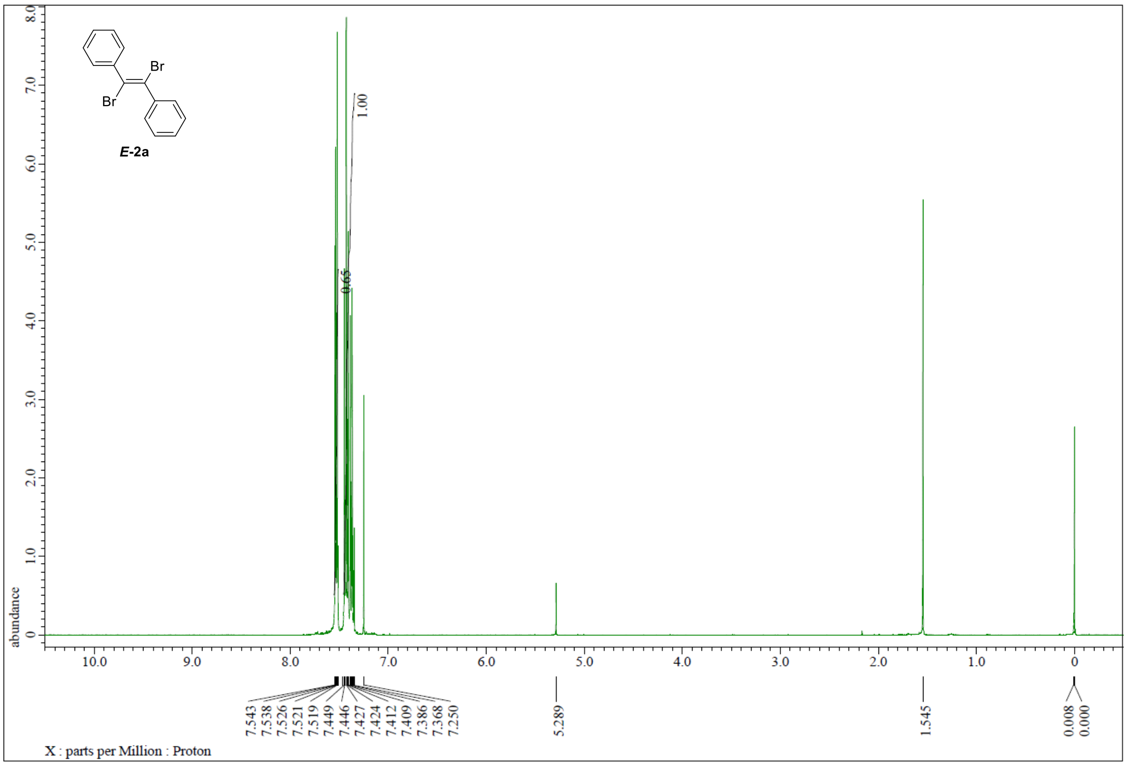

^{13}C NMR (100 MHz, CDCl3) of ***E*-2a** (Scheme 2 (a))

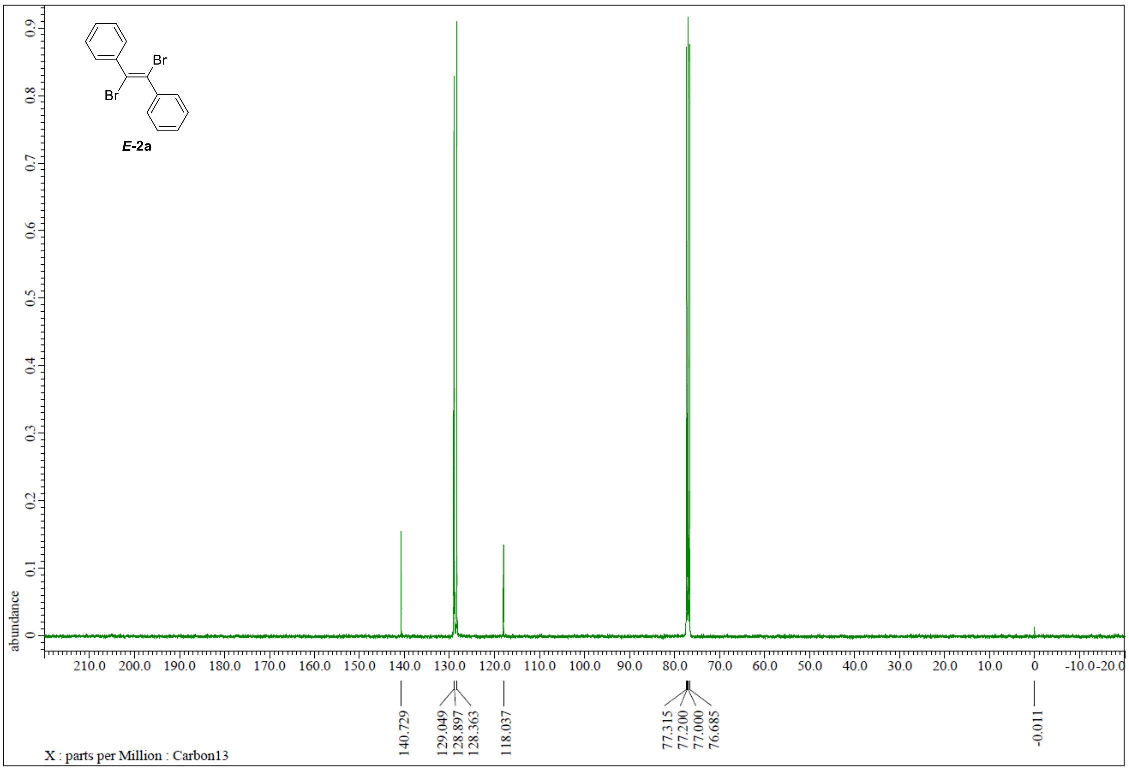

^{1}H NMR (300 MHz, $CDCl_3$) of ***Z*-2a** (Scheme 2 (a))

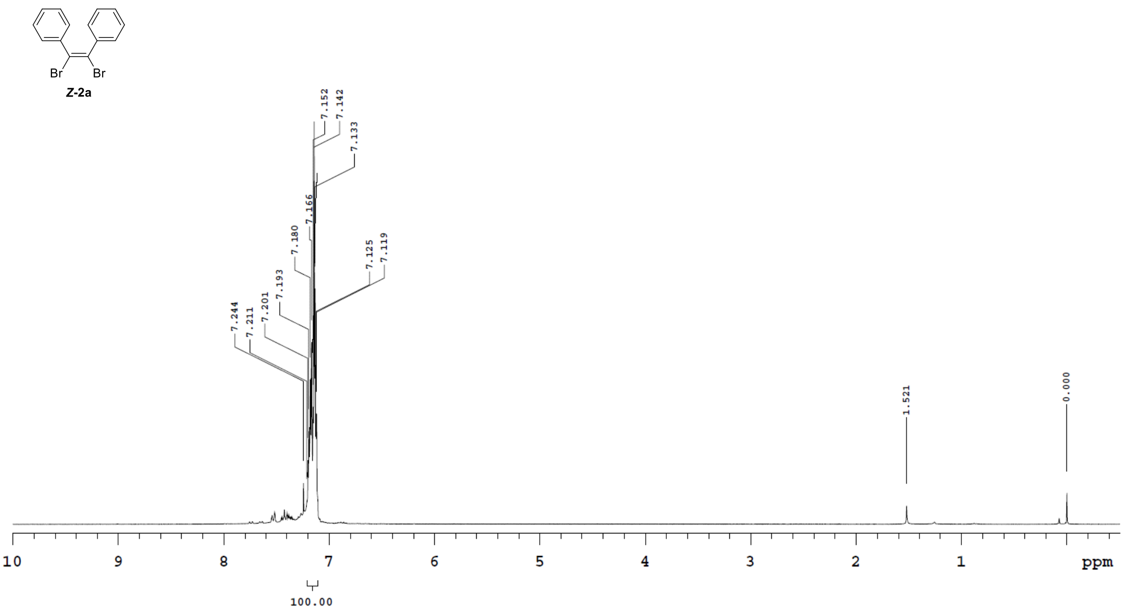

^{13}C NMR (75 MHz, $CDCl_3$) of ***Z*-2a** (Scheme 2 (a))

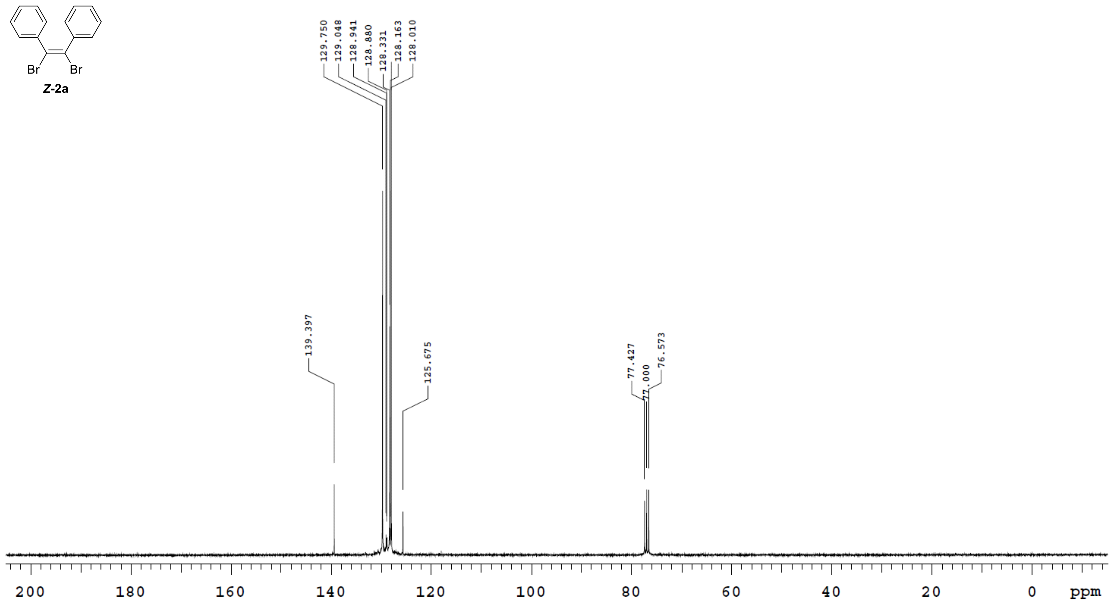

Supplement: File 1 — General remarks, preparation of substrates, experimental procedure, characterization data of compounds, and copies of 1H and 13C NMR spectra. [file Beilstein_J_Org_Chem-22-795-s001.pdf]
